# CLDN6 Expression Plasticity in Ovarian Cancer: Insights into Therapeutic Optimization for CLDN6-Targeted Immunotherapy

**DOI:** 10.1158/2767-9764.CRC-25-0399

**Published:** 2026-02-25

**Authors:** Naoki Kimura, Kenji Taniguchi, Shinichi Onishi, Chie Kato, Etsuko Fujii, Nami Yabuki, Hiromichi Terashima, Takayuki Kamikawa, Moe Yoshimoto, Atsuhiko Kato, Takehisa Kitazawa

**Affiliations:** Research Division, Chugai Pharmaceutical, Yokohama, Japan.

## Abstract

Epithelial ovarian cancer (EOC) represents the most lethal gynecologic malignancy, characterized by extensive tumor heterogeneity that contributes to treatment resistance and high recurrence rates. Recently, we developed SAIL66, a CLDN6-targeting T-cell engager currently in clinical evaluation for CLDN6-positive solid cancers, including EOC. Whereas CLDN6 is considered an attractive target for cancer therapy due to its cancer specificity, its biology remains poorly understood. In this study, we investigated the biological characteristics of CLDN6-positive EOC to identify its significance as a therapeutic target for ovarian cancer treatment. We demonstrated heterogeneous CLDN6 expression in xenograft and clinical tumors. *In vitro*–cultured ovarian cancer cell lines showed reversible changes in CLDN6 expression depending on cell density, accompanied by alterations in epithelial–mesenchymal transition (EMT)-related and stemness-related genes. Spatial transcriptomic analysis of clinical specimens revealed that CLDN6-positive areas formed both solid regions and dispersed small clusters within the same tumors, with differential expression of EMT-related and cell matrix remodeling genes between these areas, consistent with our *in vitro* observations at varying cell densities. Furthermore, carboplatin treatment increased CLDN6 expression, accompanied by changes in EMT-related genes. Leveraging these biological characteristics of CLDN6, we discovered that significant tumor regression was observed in mice treated with SAIL66 following carboplatin pretreatment. Post-carboplatin analysis revealed increased CLDN6 expression, EMT-related gene changes, and enhanced T-cell infiltration, which were associated with the synergistic effect of SAIL66. Our study provides insights into the biology and plasticity of CLDN6-positive cells in EOC heterogeneity and highlights the clinical significance of CLDN6-targeting therapies for ovarian cancer treatment.

**Significance:**

CLDN6-positive ovarian cancer cells exhibit remarkable plasticity influenced by microenvironmental factors and chemotherapy, providing critical insights for understanding the biology of ovarian cancer progression and optimizing CLDN6-targeting therapy.

## Introduction

Epithelial ovarian cancer (EOC) has the highest mortality among gynecologic cancers worldwide (1). Despite initial treatment with surgery and chemotherapy often achieving clinical remission, most patients with advanced EOC relapse within 2 years of diagnosis (2). Therefore, there is a strong demand for a deeper understanding of EOC pathology and the establishment of innovative treatment strategies.

EOC exhibits extensive tumor heterogeneity attributed to cancer stem cells (CSC) or epithelial–mesenchymal transition (EMT; refs. 3–7). Recent evidence demonstrates the plasticity of CSCs, capable of transitioning to a drug-tolerant, slow-cycling state or differentiating into multiple cellular lineages (8, 9). This plasticity explains clinical observations such as tumor recurrence after initially successful treatment as well as metastasis (10–12). EMT represents another aspect of EOC malignancy, associated with high plasticity, chemoresistance, and peritoneal dissemination. Studies have shown that EMT endows epithelial cells with CSC properties, enhancing tumor formation, migration, invasion, apoptosis prevention, and chemoresistance (13–16). Thus, targeting EMT or CSCs could offer groundbreaking therapeutic options for EOC.

CLDN6, a member of the claudin family of tight junction proteins, exhibits a unique expression profile. In normal tissues, CLDN6 expression is restricted to early development and pluripotent stem cells and coincides with the expression of the pluripotency gene OCT4 (17, 18). Its expression rapidly decreases upon differentiation into various lineages (17) and becomes undetectable in adult tissues (19–21). In contrast, CLDN6 expression is aberrantly elevated in various cancer types, including ovarian, lung, endometrial, cervical, liver, and gastric cancers, and germ cell tumors (22–32). Among them, 100% of germ cell tumors, which are characterized by primitive phenotypes, are CLDN6-positive (31). In gastric cancer, the CLDN6-positive population is exclusively classified in the “primitive phenotype” cancer category associated with poor prognosis (29).

Due to its high and specific expression in many solid tumors, CLDN6 has garnered considerable attention as a potential target for cancer therapy. Various biotherapeutic modalities such as T-cell engagers (TCEs), antibody–drug conjugates, and chimeric antigen receptor T-cell therapies targeting CLDN6 have been developed (24, 27, 33–36). Our product, SAIL66, a next-generation TCE targeting CLDN6, is also currently undergoing clinical development to evaluate efficacy and safety in patients with CLDN6-positive solid cancers (33).

Beyond its roles as a tight junction molecule, recent studies have demonstrated the significance of CLDN6 in cancer progression. CLDN6 expression is associated with a worse prognosis in many types of cancer (22–26, 28–30, 32). Gene silencing or overexpression experiments have indicated that CLDN6 promotes cancer cell proliferation, migration, and invasion in cell lines derived from hepatocellular carcinoma (27, 37, 38), gastric cancer (28), endometrial cancer (39), and choriocarcinoma (40) and is associated with EMT phenotypes.

Although these findings suggest important roles for CLDN6 in cancer progression, its significance in EOC has not been thoroughly explored. In this study, we investigated the biological characteristics of CLDN6 in EOC using *in vitro* cultured cell lines and clinical specimens. Our results demonstrate the high plasticity of EOC, characterized by dynamic changes in CLDN6 expression accompanied alterations in EMT and stemness-related genes in response to various conditions, including cellular density, TGFβ stimulation, and chemotherapeutic treatment. Based on this biology, we tested the combination effect of SAIL66, a CLDN6-targeting TCE, and chemotherapies in xenograft ovarian cancer models. Importantly, significant tumor regression was observed in mice treated with SAIL66 following carboplatin pretreatment.

Our findings provide novel insights into the biology and plasticity of CLDN6-positive cells in EOC, enhancing our understanding of tumor heterogeneity. Moreover, our findings suggest that combination strategies with chemotherapy may optimize CLDN6-targeting immunotherapy, informing clinical development to improve treatment outcomes for patients with ovarian cancer.

## Materials and Methods

### Cell lines and cell culture

To analyze the cellular characteristics, we selected seven serous ovarian cancer cell lines, as a previous study demonstrated that serous adenocarcinomas show the most frequent expression of CLDN6 among ovarian adenocarcinomas (31): OV-90 (RRID: CVCL_3768, purchased in May 2012 from the ATCC), NIH:OVCAR-3 (RRID: CVCL_0465, purchased in November 2019 from the ATCC), COV362 [RRID: CVCL_2420, purchased in September 2017 from the European Collection of Animal Cell Cultures (ECACC)], COV318 (RRID: CVCL_2419, purchased in July 2018 from the ECACC), COV413A (RRID: CVCL_2422, purchased in September 2017 from the ECACC), COV413B (RRID: CVCL_2423, purchased in September 2017 from the ECACC), and JHOS4 (RRID: CVCL_4649, purchased in June 2012 from RIKEN BRC). We included a lung adenocarcinoma cell line, NCI-H1435 (RRID: CVCL_1470, purchased in January 2014 from the ATCC), as a comparative CLDN6-positive cancer of a different tissue origin. SK-OV-3 (RRID: CVCL_0532, purchased in September 2004 from the ATCC) was used for generation of CLDN6-overexpressing transfectant to evaluate the specificity of an in-house–generated rabbit anti-human CLDN6 antibody. All cell lines were authenticated by the ATCC, ECACC, or RIKEN BRC using short tandem repeat profiling. Cell line information, including their suppliers, origin, and molecular subtypes, used in this study is listed in Supplementary Table S1.

OV-90 was maintained in a 1:1 mixture of MCDB 105 medium and Medium 199 with 15% fetal bovine serum (FBS). NIH:OVCAR-3 was grown in RPMI 1640 supplemented with 20% FBS, 4.5 g/L glucose, 10 mmol/L HEPES, 1 mmol/L sodium pyruvate, and 10 μg/mL insulin. SK-OV-3 was maintained in McCoy’s 5a medium supplemented with 10% FBS, 1.5 mmol/L glutamine, and 2.2 g/L sodium bicarbonate. COV362, COV318, COV413A, COV413B, and NCI-H1435 were maintained in Dulbecco’s modified Eagle medium (DMEM) supplemented with 10% FBS. JHOS4 was maintained in a 1:1 mixture of DMEM and Ham’s F12 supplemented with 10% FBS. Cells were maintained in logarithmic growth phase and passaged weekly or twice weekly in a humidified incubator at 37°C with 5% CO_2_. Cell lines were used within 15 passages from frozen stocks to maintain genetic authenticity, and no reauthentication was performed beyond the initial authentication by the cell banks. PCR-based *Mycoplasma* testing is routinely performed. The latest dates of testing are as follows (OV-90: May 15, 2024; NIH:OVCAR-3: September 25, 2024; COV362: April 24, 2024; COV318: September 24, 2025; COV413A: March 5, 2025; COV413B: December 14, 2022; JHOS4: not done due to limited availability; NCI-H1435: June 7, 2023; and SK-OV-3: September 24, 2025).

### Assaying cell density–dependent changes in cellular characteristics

To analyze changes in cell density–dependent cellular characteristics, cells were cultured under the following conditions: Cells were detached, counted, and then seeded into six-well plates at densities of 4 × 10^4^ cells/well (low density), 1.6 × 10^5^ cells/well (medium density), and 4 × 10^5^ cells/well (high density). After 1 week of culture, cells were harvested and used for gene expression analysis, protein expression analysis, and flow cytometry. Additionally, cells initially seeded at high density were further seeded into six-well plates at densities of 4 × 10^4^ cells/well, 1.6 × 10^5^ cells/well, and 4 × 10^5^ cells/well. These cells were harvested after 1 week and used for expression analyses.

### Chemotherapeutic drug treatment *in vitro*

NIH:OVCAR-3 cells were plated at 2.5 × 10^5^ cells/well on six-well plates. The following day, cells were treated with 0.5 μg/mL of carboplatin (SANDOZ), 0.1 μg/mL of cisplatin (Nichi-Iko), 10 ng/mL of doxorubicin (SANDOZ), and 1.2 ng/mL of paclitaxel (Nippon Kayaku). COV318 cells and COV362 cells were plated at 2.5 × 10^5^ cells/well, and NCI-H1435 cells were plated at 3 × 10^5^ cells/well on six-well plates. Medium-only wells served as nontreated controls. The following day, cells were treated with carboplatin (SANDOZ) at 2.5 μg/mL for COV318, 10 μg/mL for COV362, and 6 μg/mL for NCI-H1435 cells. After culturing the cells for 5 days in the presence of chemotherapeutic agents, the medium was changed to fresh medium. The cells were then harvested 3 or 4 days later. Gene expression analysis was subsequently performed.

### Assaying the effect of TGFβ1 on CLDN6 induction

For FACS analysis, cells were plated at a density of 5 to 6 × 10^5^ cells in T-25 flasks and incubated with/without 10 ng/mL of human TGFβ1 (R&D, 240-B). After 4 days of incubation, cells were harvested and used for FACS analysis. For qPCR, NIH:OVCAR-3 cells were plated at a density of 1.5 × 10^5^ cells/well in six-well plates in the presence or absence of 10 ng/mL of human TGFβ1. After 5 days of incubation, cells were harvested and used for qPCR.

For assaying the TGFβ-blocking effect on chemotherapy-induced *CLDN6 *expression, NIH:OVCAR-3 cells were plated at a density of 2 × 10^5^ cells/well in six-well plates. The following day, cells were treated with 3 μg/mL of carboplatin (SANDOZ), 0.5 μg/mL of irinotecan (SANDOZ), and 10 ng/mL of human TGFβ1 (R&D, 240-B). Medium-only wells served as nontreated controls. The cells were then cultured in the presence or absence of 100 μg/mL of anti-TGFβ antibody (Bio X Cell, BE0057, RRID: AB_1107757).

For assaying the TGFβ-blocking effect on *CLDN6* expression induced by low density culture, NIH:OVCAR-3 cells were plated in six-well plates at two densities: 4 × 10^4^ cells/well (low density) and 4 × 10^5^ cells/well (high density). Cells were cultured with or without 100 μg/mL of anti-TGFβ antibody.

For chemotherapy-treated cells, samples were collected after 72 hours of incubation. For density-dependent experiments, samples were collected after 6 days of culture. All samples were subsequently analyzed by qPCR.

### Gene expression analysis

For RNA extraction from tumor tissues, tumors stored in RNAlater solution (Thermo Fisher Scientific, AM7021) were homogenized using TissueLyserIII (QIAGEN). Total RNA from homogenized tissues or cultured cell lines was extracted using RNAeasy Mini Kit (QIAGEN, 74106) according to the manufacturer’s instructions.

For qPCR, RNAs isolated from tumors or cells were subjected to cDNA synthesis using Super Script IV VILO Master Mix (Thermo Fisher Scientific, 11756050) according to the manufacturer’s instructions. Primers against *CLDN6* (fw_GGGTGGACGTCTTATCAGGA, rev_GAGCTCCTCTCTTCACCCCT), *CD44* (fw_CTGGCGCAGATCGATTTGAATA TAAC, rev_TGTGGGCAAGGTGCTATTGAAAG), *OCT4* (fw_TGAGTCAGTGAAC AGGGAATG, rev_AATCTCCCCTTTCCATTCGG), *TGFβ1* (fw_AGTGGTTGAGCCGTGGAG, rev_CGGTAGTGAACCCGTTGAT), *CD3* (fw_GCAGGCAAAGGGGACAAAA, rev_TCAGATGCGTCTCTGATTCAG), and *GAPDH* (fw_CACCATCTTCCAGGAGCGAGA, rev GCA​AAT​GAG​CCC​CAG​CCTTC) were designed and synthesized by Sigma-Aldrich. qPCR was performed in a QuantStudio Real-Time PCR System (Thermo Fisher Scientific) using Power SYBR Green PCR Master Mix (Thermo Fisher Scientific, 4368577). Amplification was performed under the following conditions: one cycle of 2 minutes at 50°C and 10 minutes at 95°C and then 40 cycles of 15 seconds at 95°C and 1 minute at 60°C. Expression levels of each gene were calculated as a ratio of *GAPDH*.

For nCounter analysis, RNA was subjected to expression analysis of various immune-related genes or EMT-related genes using an nCounter PanCancer Immune Profiling Panel (NanoString Technologies, XT-CSO-HIP1-12) or an nCounter PanCancer Progression Panel (NanoString Technologies, XT-CSO-PROG1-12). The protocol was followed according to standard nCounter instructions. Sample processing and RNA counting were performed using the nCounter Prep Station and the nCounter digital analyzer (NanoString Technologies, RRID: SCR_021712). Data were normalized to yield *Z*-scores (Microsoft Excel, RRID: SCR_016137). GraphPad Prism 8.4.3 (RRID: SCR_002798) was used to generate the heatmap.

### Western blot analysis

Mouse subcutaneous transplanted tumors were rapidly frozen in liquid nitrogen after collection and stored at −80°C in a freezer until use. Frozen tumors were disrupted using Multi-beads shocker (Yasui Kikai) and used for lysate preparation. Cultured cells were washed with cold PBS before being used for lysate preparation. In both cases, samples were dissolved in Cell Extraction Buffer (Thermo Fisher Scientific, FNN0011) containing Protease Inhibitor Cocktail (Thermo Fisher Scientific, 87786) to prepare the lysates. Protein concentrations of the resulting lysates were calculated using the Bio-Rad DC protein assay kit.

Lysates (10 μg) were electrophoresed on 4% to 15% Mini-PROTEAN TGX Precast Protein Gels (Bio-Rad) and transferred to a polyvinylidene difluoride membrane using a Trans-Blot Turbo system (Bio-Rad). Blots were incubated with rabbit monoclonal anti-CLDN6 (CDB0043hh, in-house prepared) or rabbit monoclonal anti-GAPDH (Cell Signaling Technology, #2118, RRID: AB_561053) and further incubated with a horseradish peroxidase (HRP)-conjugated polyclonal goat anti-rabbit antibody (Cell Signaling Technology, #7074, RRID: AB_2099233). The series of steps including blocking, antibody incubation, and washing were performed using the Invitrogen iBind Western System (Thermo Fisher Scientific). The antibody-tagged protein bands were developed with ECL Prime Western Blotting Detection Reagent (GE Healthcare) and visualized with the ChemiDoc Gel Imaging System (Bio-Rad, RRID: SCR_019684). The density of CLDN6 band in Western blot was quantified using the Image Lab Software (RRID: SCR_014210),

### Flow cytometry

Cells were dissociated with Cell Dissociation Reagents Accutase (Nakalai Tesque) and resuspended in FACS buffer consisting of autoMACS Rinsing Solution (Miltenyi Biotec, #130-091-222) supplemented with 0.5% bovine serum albumin (BSA; Miltenyi Biotec, #130-091-376). The following antibodies were used for flow cytometric analysis: CD44 monoclonal antibody (IM7)-APC (Thermo Fisher Scientific, #17-0441-82, RRID: AB_469390) and human claudin-3 APC (R&D Systems, FAB4620A, RRID: AB_1207861). For CLDN6 detection, a custom antibody (SAIL66-mIgG) was generated by fusing the Fab region of SAIL66 to the Fc region of mouse IgG1, which was subsequently conjugated with Alexa Fluor 488 using the Alexa Fluor 488 Antibody Labeling Kit (Thermo Fisher Scientific, A20181) according to the manufacturer’s protocol. Cells were incubated with fluorescent-labeled antibodies in FACS buffer at the following concentrations: 10 μg/mL of anti-CLDN6, 1:100 dilution of anti-CD44, and 1:15 dilution of anti-CLDN3, for 30 to 40 minutes at 4°C in the dark. Following two washes with FACS buffer, flow cytometry analysis was performed using the BD FACSLyric Flow Cytometry System (BD Biosciences). Cells were initially gated based on forward scatter (FSC) and side scatter (SSC) properties, followed by doublet exclusion using FSC-W versus FSC-H and SSC-W versus SSC-H plots. Dead cells were excluded using Fixable Viability Dye eFluor 780 (Thermo Fisher Scientific, 65-0865-14). For CLDN6 and CD44 expression analysis, appropriate isotype controls were used to establish positive gates. The “CLDN6^high^/CD44^high^” population was defined as cells showing fluorescence intensity above the 98th percentile of the respective isotype control for both markers. The gating strategy is shown in Supplementary Fig. S1A and S1B. Data were analyzed using FlowJo 10 software (BD Biosciences, RRID: SCR_008520), and the results are presented as mean fluorescence intensity (MFI) values or the percentage of CLDN6^high^/CD44^high^ population.

### Immunofluorescence microscopy

NIH:OVCAR-3 cells were seeded into 24-well plates at densities of 9 × 10^3^ cells/well (low density) and 9 × 10^4^ cells/well (high density). After 5 days of culture, cells were fixed with 4% paraformaldehyde in PBS at room temperature, permeabilized with 0.1% Triton X-100 in PBS, and blocked with 1% BSA in PBS-T (PBS containing 0.05% Tween 20). The cells were double-stained with rabbit anti-human CLDN6 antibody (clone CDB0324ff, Chugai Pharmaceutical, Co., Ltd.) and mouse anti-Ki-67 antibody, 37C7-12 (Abcam, ab245113, RRID: AB_2923193) for 1 hour at room temperature. The cells were then incubated with anti-rabbit IgG, Alexa Fluor plus 488 (Thermo Fisher Scientific, A-11008, RRID: AB_143165) for CLDN6 detection and anti-mouse IgG, Alexa Fluor plus 647 (Thermo Fisher Scientific, A32728TR, RRID: AB_2866490) for Ki-67. Hoechst 33342 (Thermo Fisher Scientific, H3570) was used for nuclear staining. Immunofluorescence images were acquired using a BZ-X800 microscope (Keyence, RRID: SCR_023617).

### *In vivo* antitumor efficacy study

Humanized NOG (huNOG) mice used for all *in vivo* studies were generated as described below. NOD/Shi-scid/IL-2Rγnull (NOG) mice (*in vivo* science) were irradiated (2.5 Gy) 1 day before transplantation. Human hematopoietic stem cells (HSC) from cord blood (Lonza) were intravenously injected into NOG mice (approximately 1 × 10^5^ cells/mouse). After transplantation of HSCs, human T-cell chimerism in mice was confirmed by assessing the presence of human CD45-positive and human CD3-positive cells in blood.

OV-90 (5 × 10^6^ cells/mouse) or NIH:OVCAR-3 (1 × 10^7^ cells/mouse) cancer cells were implanted subcutaneously into the right flanks of the mice with Matrigel Basement Membrane Matrix (Corning). When the tumor size reached 150 to 200 mm^3^, animals were randomized into appropriate groups for the following studies.

#### Study for SAIL66 monotherapy

Mice were randomized into two groups of eight animals each (day 0) and then treated with PBS containing 0.05% Tween 20 as a vehicle (Sigma Aldrich) or SAIL66 at 1 mg/kg once.

#### Study for combination therapy I

Mice were randomized into five groups of five animals each (day 0) and then treated with isotype control IgG (0.3 mg/kg), carboplatin (30 mg/kg), and SAIL66 (0.3 mg/kg) alone on days 0 and 7. In the concurrent treatment group, mice received carboplatin and SAIL66 on days 0 and 7. In the sequential treatment group, carboplatin was administered on days 0 and 7 first, and then SAIL66 was administered on days 10 and 17 (Supplementary Fig. S2A).

#### Study for combination therapy II

Mice were randomized into six groups of five animals each (day 0) and then treated with/without two cycles of cytotoxic agents (30 mg/kg of carboplatin or 10 mg/kg of doxil; days 0 and 7). After the pretreatment of cytotoxic agents, mice were treated with isotype control IgG (0.3 mg/kg) or SAIL66 (0.3 mg/kg) twice (days 10 and 17; Supplementary Fig. S2B).

#### Study for pharmacodynamics analysis after carboplatin administration

Mice were randomized into four groups of five animals each and administered saline (vehicle) or carboplatin (30 mg/kg) on days 0 and 7. Tumors were harvested 3 and 10 days after the second carboplatin administration and used for studies.

SAIL66, isotype IgG, and doxil (Janssen Pharmaceutical K.K.) were administered intravenously, and vehicle and carboplatin (Bristol Myers Squibb) were administered intraperitoneally. Tumors and body weight were measured twice each week, and tumor volumes were calculated using the formula [(length × width^2^)/2]. Xenograft tumor tissues were fixed in 10% neutral buffered formalin and embedded into paraffin by a routine method.

### Immunohistochemical staining

For the analysis of CLDN6 protein expression in formalin-fixed, paraffin-embedded (FFPE) samples, immunohistochemistry (IHC) was performed. The specificity of in-house–generated rabbit anti-human CLDN6 antibody (clone CDB0324ff, Chugai Pharmaceutical, Co., Ltd.) was confirmed in FFPE cell blocks by comparing CLDN6-transfected cells with parental cells (Supplementary Fig. S3A and S3B).

Briefly, sections were cut at 3 to 4 μm thickness. After deparaffinization, antigen retrieval was performed using an antigen retrieval solution (S236784-2, Agilent Technologies) by heating with a microwave tissue processor (LP2850, Energy Beam Science, East Granby, CT, USA) at 98°C for 15 minutes. Endogenous peroxidase activity was quenched with 0.3% H_2_O_2_ in methanol at room temperature for 30 minutes, followed by blocking with 5% BSA in 0.05 mol/L TBS at room temperature for 30 minutes. The CLDN6 antibody was applied as the primary antibody at a concentration of 5 μg/mL and incubated overnight at 4°C. Slides were then incubated with a secondary antibody (horse anti-rabbit IgG polyclonal antibody, polymer-HRP, MP-6401, Vector Laboratories) at room temperature for 30 minutes and visualized with 3,3-diaminobenzidine tetrahydrochloride solution (FUJIFILM Wako Pure Chemical Corporation) in 0.05 mol/L Tris-HCl buffer (pH 7.6) with 0.03% H_2_O_2_. The slides were counterstained with hematoxylin and coverslipped for examination under a light microscope. CLDN6 expression level was quantified using the H-score, calculated from DAB staining intensity of tumor cells within each tissue, ranging from 0 to 300 (41). Scores were calculated using the following formula: H-score = (1 × percentage of weak staining) + (2 × percentage of moderate staining) + (3 × percentage of strong staining). The tissues were examined and scored independently by two pathologists.

For the analysis of CD3-positive cells, FFPE samples were sectioned as described above. IHC for human CD3 (clone 2GV6, Roche Diagnostics K.K., Rotkreuz, Switzerland, RRID: AB_2335978) was performed using the OptiView DAB IHC Detection Kit (Roche Diagnostics K.K) on a VENTANA BenchMark XT autostainer (Roche Diagnostics K.K, RRID: SCR_026335).

### GeoMx spatial transcriptomics

Spatial transcriptomics was performed using a Nanostring GeoMx Digital Spatial Profiler (DSP; RRID: SCR_021660). After deparaffinization of the slides, target retrieval was performed by incubating in Tris-EDTA at 99°C for 15 minutes. To expose RNA targets, samples were incubated with 1 μg/mL proteinase K at 37°C for 15 minutes. Following post-fixation, samples were incubated overnight with whole-transcriptome atlas probes. After washing, the slides were stained with SYTO83, pan-cytokeratin (PanCK), and CLDN6 (clone CDB0324ff, Chugai Pharmaceutical, Co., Ltd.) for 100 minutes. Slides were scanned using GeoMx DSP, and 4 to 5 regions of interest (ROI) were selected for both solid and small cluster areas with high CLDN6 expression. PanCK was used for segmentation, and barcode oligoes were collected from PanCK+ and PanCK− regions separately by UV cleavage to a collection plate. The collected oligoes then were uniquely indexed and amplified by PCR using GeoMx Seq Code Pack (GMX-NGS-SEQ-GH) to obtain next-generation sequencing (NGS) libraries. The NGS libraries were paired-end sequenced (2 × 75) on a NextSeq500 system (Illumina). Fastq files were further processed in GeoMx NGS pipeline software, and generated DCC files were uploaded to the GeoMx DSP instrument. Using GeoMx DSP data analysis software, data processing and Q3 normalization were performed, followed by analysis of gene expression for each gene. Differential expressed genes between small clusters and solid areas were analyzed.

### Pathway analysis using IPA

Pathway analysis was performed using IPA (QIAGEN bioinformatics, https://digitalinsights.qiagen.com/products/ingenuity-pathway-analysis). The cutoff was set at *P* value < 0.05 (42).

### Quantification and statistical analyses

GraphPad Prism version 10 was used for the graphical representation of data. Data are expressed as the mean ± SD. All experiments were repeated at least in triplicate. The statistical differences were examined by two-tailed unpaired Student *t* test or Dunnett test, as indicated in the figure legend. The results were considered statistically significant when the *P* value was less than 0.05.

### Ethical approval and consent to participate

All *in vivo* mouse experimental procedures were approved by the Institutional Animal Care and Use Committee of Chugai Pharmaceutical, in compliance with the Japanese Guidelines for Proper Conduct of Animal Experiments, Standards relating to the Care and Keeping and Reducing Pain of Laboratory Animals, and The Act on Welfare and Management of Animals. Chugai Pharmaceutical is accredited by the Association for the Assessment and Accreditation of Laboratory Animal Care International. Mice were housed in microisolator units maintained on a 12-hour light/dark cycle. Mice were given standard chow and water. The use of human materials was approved by the Institutional Review Board of Chugai Pharmaceutical in accordance with the Declaration of Helsinki. Human HSCs collected from donors with written informed consent were purchased from Lonza. FFPE clinical ovarian cancer tissues from patients with written informed consent were purchased from ProteoGenex.

## Results

### CLDN6 exhibits heterogeneous expression in ovarian tumors

**Figure 1.**
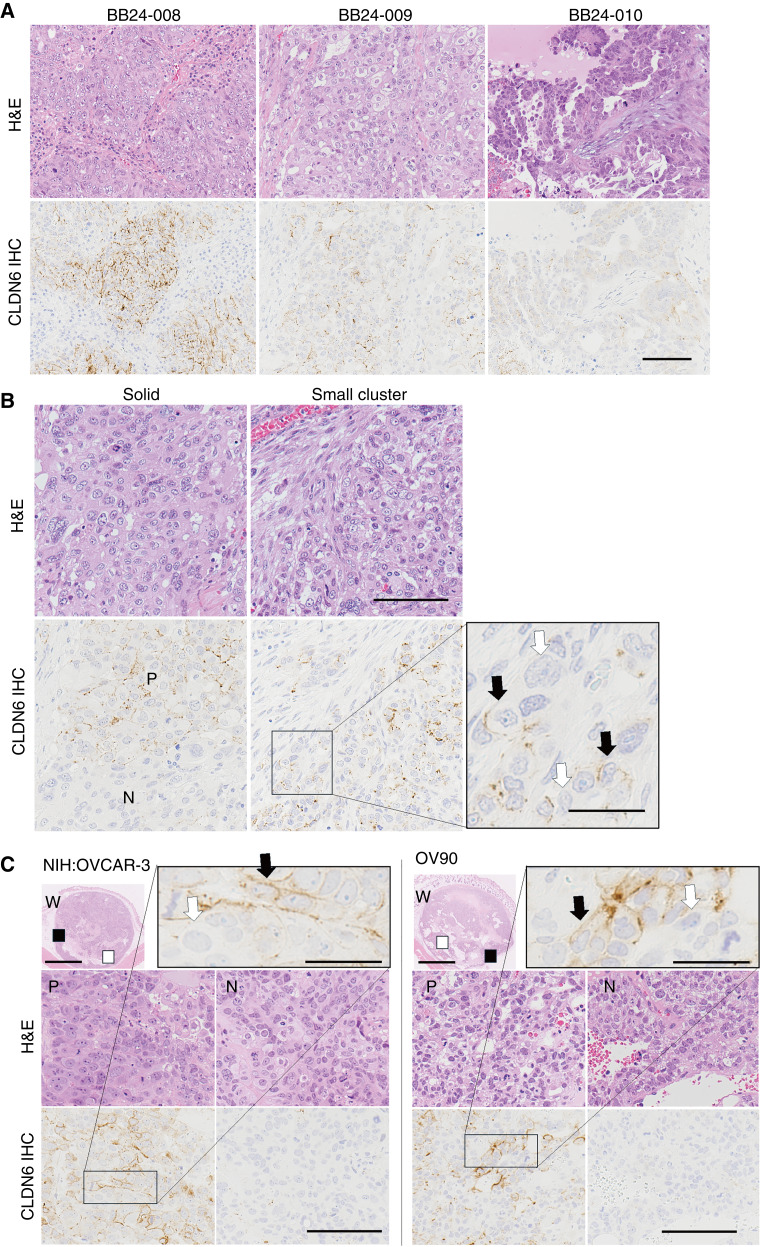
Heterogeneity of CLDN6 expression in clinical ovarian serous adenocarcinoma and xenograft tumor tissue. **A,** Interpatient heterogeneity of CLDN6 expression in clinical serous ovarian adenocarcinoma. The positive staining frequency of tumor cells varied among cases. Scale bar, 100 μm. **B,** Intratumor heterogeneity of CLDN6 expression in clinical serous ovarian carcinoma (BB24-009). Positive (P) and negative (N) areas are observed within the same tissue, both in solid tumor areas (solid) and areas with small tumor cell clusters (small cluster). Positive (black arrows) and negative (white arrows) tumor cells exist in the same area. Scale bar, 100 μm (insert, 30 μm). **C,** Intratumor heterogeneity of CLDN6 expression in two different ovarian cancer cell xenograft tumor tissues, NIH:OVCAR-3 and OV-90. Positive (P, black squares) and negative (N, white squares) areas are seen within the same tissue, as well as positive (black arrows) and negative (white arrows) cells within the same areas (inserts). CLDN6 IHC, IHC staining for CLDN6; H&E, hematoxylin and eosin stain. Scale bars, 100 μm (whole tissue, 2.5 mm; insert, 30 μm).

To investigate the expression of CLDN6 in ovarian cancer, we analyzed clinical tissue samples from seven patients with ovarian serous adenocarcinoma using IHC staining. Previous studies have reported intratumoral heterogeneity of CLDN6 protein in serous ovarian cancer (22) and endometrial cancer tissues (25). In our study, despite the limited sample size, five of seven cases were positive for CLDN6, with varying expression levels among patients. H-scores ranged from 0 to 143, suggesting substantial interpatient heterogeneity (Fig. 1A; Supplementary Table S2). Within the CLDN6-positive tissues, we observed distinct positive and negative areas as well as positive and negative cells adjacent to each other (Fig. 1B), confirming intratumoral heterogeneity consistent with previous reports (22, 25). Notably, CLDN6-positive cells in clinical specimens were localized to two morphologically distinct areas: tumor cells forming nests (solid, in Fig. 1B) and small tumor cell clusters with abundant stroma (small cluster, in Fig. 1B). Additionally, CLDN6 expression within the positive areas showed extremely high heterogeneity. These findings demonstrate notable intertumoral and intratumoral heterogeneity in CLDN6 expression even within this limited cohort. Based on these observations, we sought to elucidate the biological mechanisms underlying the heterogeneity of CLDN6-positive cancer cells, which may be crucial for the development of effective CLDN6-targeting therapeutic strategies.

### CLDN6 expression in ovarian cancer cell lines exhibits high plasticity, showing heterogeneous expression in subcutaneous xenograft tumors

**Figure 2.**
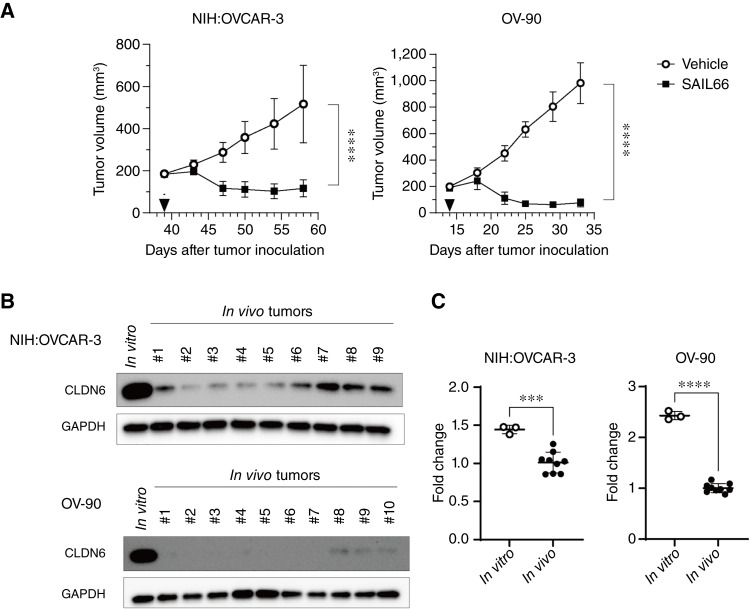
In xenograft tumors that show strong response to SAIL66 administration, the expression of CLDN6 is significantly reduced under vivo growth conditions. **A,** Tumor growth inhibition by SAIL66 (1 mg/kg) in huNOG mice bearing NIH:OVCAR-3 and OV-90 xenografts compared with vehicle-treated control group (*n* = 8 per group). **B,** Western blot analysis of CLDN6 protein levels in NIH:OVCAR-3 and OV-90 cultured *in vitro* and xenograft tumors established in mice (*in vivo*). **C,** qPCR analysis of *CLDN6* gene expression in NIH:OVCAR-3 and OV-90 cultured *in vitro* and tumors established in mice (*in vivo*). Quantitative data are presented as the mean ± SD. Two-tailed Student *t* test. ***, *P* < 0.001; ****, *P* < 0.0001.

SAIL66, a novel CLDN6-targeting next-generation TCE, is currently undergoing clinical evaluation for CLDN6-positive solid cancers, including ovarian cancer (33). A single administration of SAIL66 demonstrated potent antitumor activity against NIH:OVCAR-3 and OV-90 tumors in humanized (huNOG) mice xenograft models (Fig. 2A), supporting CLDN6 as a promising target for TCEs. Flow cytometric analysis of *in vitro*–cultured OV-90 (Supplementary Fig. S4A) and NIH:OVCAR-3 (Supplementary Fig. S4B) cells revealed high-level CLDN6 expression with a single peak. However, IHC staining of these xenograft tumors revealed the intertumoral heterogeneity of CLDN6 expression (Fig. 1C), similar to clinical tumors (Fig. 1A and B).

Interestingly, CLDN6 expression in cultured cells was markedly decreased in xenografted tumors at both protein and RNA levels (Fig. 2B and C). These findings suggest that CLDN6-positive cells exhibit a high degree of phenotypic plasticity.

### A sparse cellular state leads to increased expression of CLDN6, accompanied by EMT and dedifferentiation

Previous reports indicate that CLDN6 expression is primarily limited to undifferentiated cells such as embryonic stem cells (18, 43) and pluripotent stem cells (44) or is associated with (EMT in hepatocellular carcinoma (27, 37, 38) and gastric cancer (28). Based on these findings, we hypothesized that the plasticity observed in CLDN6-positive cancer cells might be attributed to cellular differentiation states or associated with EMT processes.

**Figure 3.**
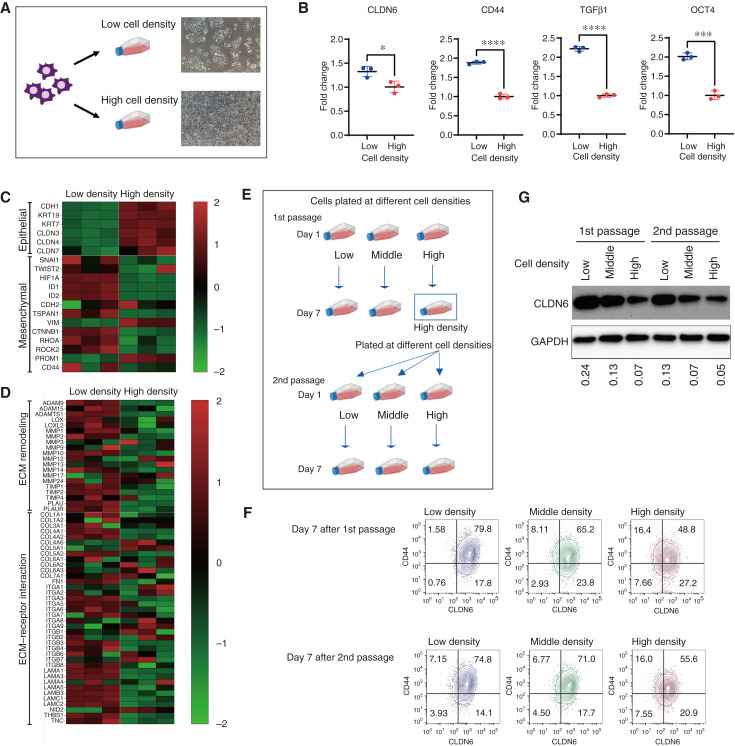
CLDN6 expression reversibly fluctuates *in vitro* depending on cell density, accompanied by changes in EMT-, ECM-, and stem cell–related genes. **A,** Schematic of *in vitro* culture experiments with different cell densities. NIH:OVCAR-3 cells were plated at low or high density for 1 week and used for gene expression analysis. Brightfield microscopy images show the cell confluency of NIH:OVCAR-3 cells after 7 days of culture at low and high cell densities. **B,** qPCR analysis of indicated gene expression in NIH:OVCAR-3 cells grown at low and high cell density for 7 days. Quantitative data are presented as the mean ± SD (*n* = 3). Two-tailed Student *t* test. *, *P* < 0.05; ***, *P* < 0.001; ****, *P* < 0.0001. **C** and **D,** Heatmap of nCounter Analysis System results for NIH:OVCAR-3 cells grown at low or high cell density for 7 days (*n* = 3). The heatmap shows differential expression of genes involved in EMT (**C**) and ECM remodeling and ECM–receptor interaction (**D**) between cells with low and high cell densities. **E,** Schematic of *in vitro* culture experiments with reexpansion of NIH:OVCAR-3 cells at high cell density. On day 1, NIH:OVCAR-3 cells are plated at low, middle, and high cell densities. After 1 week of culture, cells are used for gene expression analysis (day 7 after first passage). Cells initially plated at high density are further seeded at low, middle, and high cell densities again. These cells were harvested after one additional week and used for expression analyses (day 7 after second passage). **F,** Representative flow cytometry plots of NIH:OVCAR-3 cells after first and second passages with different cell densities, assessed for the expression of CLDN6 and CD44. The plots of cells stained with isotype control are shown in Supplementary Fig. S6. **G,** Western blot analysis of CLDN6 protein levels in NIH:OVCAR-3 cells. NIH:OVCAR-3 cells were plated at low, middle, and high cell densities, and CLDN6 expression was examined after the first 7-day culture (first passage) and second 7-day culture (second passage). Densitometric analysis of CLDN6 normalized to GAPDH is shown below each lane.

To test this hypothesis, we examined CLDN6 expression in cells grown at varying cell densities, which influence cellular differentiation and EMT status (45–47). We cultured NIH:OVCAR-3 cells at low and high densities for 7 days and quantified the expression level of *CLDN6*, *CD44* (a CSC marker; ref. 7), and *OCT4* (a stem cell marker). RT-qPCR analysis revealed significantly higher expression of *CLDN6*, *CD44*, *TGFβ1*, and *OCT4* in cells grown at low density compared with high density (Fig. 3B).

We further assessed changes in EMT-related gene expression between cells grown at low and high density using a NanoString nCounter PanCancer Progression Panel. Gene expression profiles visualized with a heat map revealed that a sparse cellular state resulted in downregulation of epithelial-related genes and upregulation of mesenchymal-related genes (Fig. 3C). Additionally, cells grown at low cell density showed upregulation of extracellular matrix (ECM) remodeling and ECM–receptor interaction–related genes (Fig. 3D), indicating a shift toward a more mesenchymal-like phenotype under sparse cellular conditions.

To determine whether upregulation of CLDN6 expression in low-density cultures was cell cycle–independent, we analyzed the expression of CLDN6 and Ki-67 (a proliferation marker reaching peak levels during mitosis; ref. 48) in NIH:OVCAR-3 cells at both low and high cell densities. Immunocytochemistry revealed no correlation between these proteins (Supplementary Fig. S5A and S5B), suggesting that density-dependent regulation of CLDN6 occurs independently of cell-cycle progression. Notably, CLDN6 localization differed based on cellular context: it was predominantly localized at cell boundaries in cohesive clusters allowing proper formation of tight junctions (white arrows and white arrowheads in Supplementary Fig. S5A and S5B) but showed strong intracellular expression with minimal border localization in less adhesive cells at cluster peripheries in low-density cultures (yellow arrowheads in Supplementary Fig. S5A).

Collectively, these findings suggest that the CLDN6-positive population exhibits significant phenotypic plasticity. This plasticity seems to be independent of cell-cycle progression but may be closely associated with cellular density and adhesion status.

### The population of cells expressing high levels of CLDN6 and CD44 reversibly fluctuates depending on cell density

To examine whether cell density–dependent changes in the CLDN6-positive population are reversible, NIH:OVCAR-3 cells grown at overconfluence were allowed to reexpand in culture at different cell densities (Fig. 3E). The proportion of the CLDN6-positive population was then assessed by flow cytometry after double-staining the cells with CLDN6 and CD44 antibodies. Flow cytometry scatter plots of NIH:OVCAR-3 cells showed coexpression of CLDN6 and CD44. On day 7 after the first passage, the CLDN6^high^/CD44^high^ population was 79.8% in cells grown at low cell density, whereas it was 48.8% in cells grown at high cell density. Interestingly, when the overconfluent cells were replated at low density, the CLDN6^high^/CD44^high^ population increased to approximately 75% after 7 days of reexpansion (Fig. 3F; Supplementary Figs. S6 and S7A). This cell density–dependent reversible change in CLDN6 expression was also confirmed by Western blotting (Fig. 3G). These results suggest that the CLDN6^high^/CD44^high^ population may arise *de novo* from the CLDN6^low^/CD44^low^ population in a cell density–dependent manner.

As CLDN3 has been reported to have increased expression in ovarian cancer and an important role in tumor growth and metastasis (49, 50), we evaluated CLDN3 expression in NIH:OVCAR-3 cells. Flow cytometry analysis showed that NIH:OVCAR-3 cells expressed high levels of CLDN3, and CLDN3 expression was increased under higher cell density conditions (Supplementary Fig. S7B), unlike CLDN6 and CD44.

We further confirmed the cell density–dependent changes in the CLDN6-high population using four additional ovarian cancer cell lines (COV362, COV413B, JHOS4, and OV-90) and a lung adenocarcinoma cell line (NCI-H1435), which were confirmed to be CLDN6 expression–positive at varying levels beforehand (Supplementary Fig. S4A and S4C–S4G). Flow cytometry scatter plots showed that all ovarian and lung adenocarcinoma cells coexpressed CLDN6 and CD44, and the CLDN6^high^/CD44^high^ population of all cells (Supplementary Fig. S8A–S8D and S8F–S8I), except for OV-90 (Supplementary Fig. S8E and S8J), varied in a cell density–dependent manner, as observed in NIH:OVCAR-3 cells.

### Gene expression patterns differed between morphologically distinct CLDN6-positive areas within clinical ovarian cancer specimens

In clinical specimens, CLDN6-positive cells formed two morphologically distinct areas within the same tumor: solid tumor areas and dispersed small tumor areas (Fig. 1B). *In vitro*, we demonstrated that CLDN6-positive cancer cells exhibit different gene expression patterns and subcellular localization depending on culture density (Fig. 3; Supplementary Fig. S5A and S5B). These findings prompted us to investigate whether differences in gene expression patterns are also present in different CLDN6-positive areas within clinical ovarian cancer specimens.

**Figure 4.**
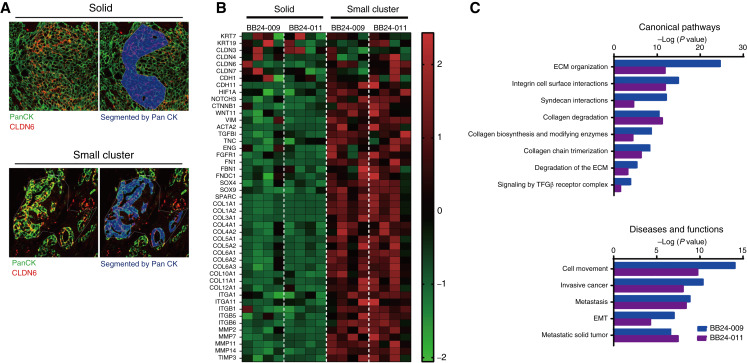
Spatial transcriptome analysis reveals distinct molecular profiles of CLDN6-positive solid tumor areas and small clusters in ovarian cancer specimens. **A,** Representative images of ovarian cancer tissue stained for CLDN6 and PanCK. Gene expression profiles were compared between four PanCK-positive ROIs from each of two identified growth patterns in ovarian cancer tissue: solid tumor areas and small clusters. **B,** Heatmap of relative gene expression per ROIs (*n* = 4) selected from two morphologically different areas (solid and small cluster) in clinical tissue samples from two patients with ovarian serous adenocarcinoma (BB24-009 and BB24-011). Small clusters showed higher expression of EMT-related genes, including *catenin beta-1* (*CTNNB1*) and *vimentin* (*VIM*) and cell matrix–related genes, including matrix metalloproteinase family (MMPs),collagen family (COLs), integrin alpha family (ITGAs), and integrin beta family (ITGBs), compared with solid areas. No significant differences were observed in epithelial markers (*KRT7* and *KRT19*) or adhesion molecules (*CLDN3*, *CLDN4*, and *CLDN7*). **C,** Pathway analysis revealed that small clusters exhibited upregulation of pathways associated with ECM remodeling, invasion and metastasis, and EMT compared with solid areas.

To this end, we performed spatial RNA profiling in human ovarian cancer tissue sections using the Nanostring GeoMx DSP. Two ovarian cancer specimens were stained with antibodies against both CLDN6 and PanCK. Next, from double-positive areas, two morphologically different areas were selected as ROIs: solid tumor areas (solid) and dispersed small tumor areas (small cluster). We then compared the gene expression profiles of PanCK-segmented areas in 4 ROIs from each area (Fig. 4A). Although this analysis was performed on a limited number of specimens, several notable differences were observed.

Among all morphologically distinct CLDN6-positive areas, the differences in *CLDN6* expression levels were not as pronounced as those observed *in vitro*–cultured cells at varying cell densities. Simultaneously, there were no significant differences in the expression of epithelial markers such as cytokeratins (*KRT7* and *KRT19*) and *CLDNs* (*CLDN3*, *CLDN4*, and *CLDN7*; Fig. 4B), indicating that all cells from the 4 ROI areas were of epithelial origin. On the other hand, CLDN6-positive areas with small clusters showed higher expression of EMT-related genes such as *catenin beta-1* (*CTNNB1*) and *vimentin* (*VIM*) and cell matrix-related genes, including matrix metalloproteinase family (MMPs), collagen family (COLs), integrin alpha family (ITGAs), and integrin beta family (ITGBs), compared with those with solid areas (Fig. 4B). Pathway analysis revealed that, compared with solid areas, small clusters demonstrated upregulation of pathways associated with ECM remodeling, invasion and metastasis, and EMT (Fig. 4C).

These results suggest that morphologically distinct CLDN6-positive areas within the same clinical tumors represent cell populations with different molecular characteristics. Furthermore, these observations suggest that the plasticity of CLDN6-positive cells observed *in vitro* may also occur in actual clinical tumors. However, given that *in vitro* models have inherent limitations in recapitulating the complexity of the *in vivo* tumor microenvironment (TME), direct extrapolation to clinical tumors should be made with caution.

### Carboplatin increases CLDN6 expression accompanied by EMT

It has been reported that chemotherapeutics induce TGFβ1 production and consequently enhance TGFβ signaling, thereby inducing EMT (51). Thus, we explored the effect of chemotherapy on *CLDN6* expression and EMT induction *in vitro*. In this study, NIH:OVCAR-3 cells were treated with chemotherapeutic agents commonly used for ovarian cancer treatment: carboplatin, cisplatin, doxorubicin, and paclitaxel. These drugs were applied at concentrations that achieved 50% of cell growth inhibition (IC_50_). After treatment, cells were harvested on day 8 for gene expression analysis.

**Figure 5.**
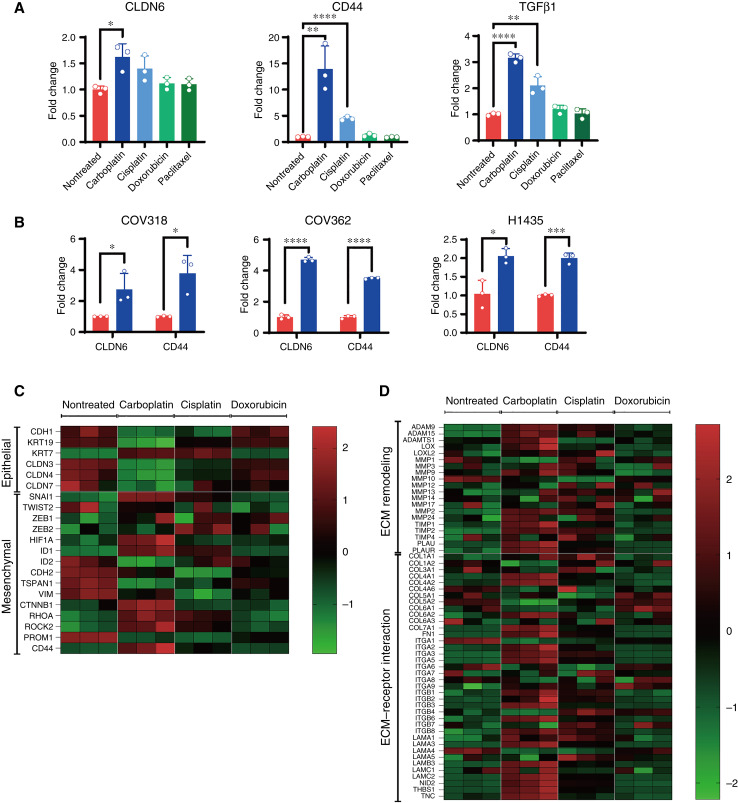
Carboplatin increases CLDN6 expression, accompanied by changes in EMT- and ECM-related genes. **A,** qPCR data of *CLDN6*, *CD44*, and *TGFβ1* expression in NIH:OVCAR-3 cells treated with indicated cytotoxic agents for 8 days. Medium-only wells served as the nontreated control. Quantitative data are presented as the mean ± SD (*n* = 3). *P* values were determined by two-tailed Student *t* test. *, *P* < 0.05; **, *P* < 0.01; ****, *P* < 0.0001. **B,** qPCR data of *CLDN6* and* CD44* in COV318, COV362, and H1435 cells treated with carboplatin (blue) or medium only (nontreated, red) for 7 or 8 days. Quantitative data are presented as the mean ± SD (*n* = 3). *P* values were determined by two-tailed Student *t* test. *, *P* < 0.05; ***, *P* < 0.001; ****, *P* < 0.0001. **C** and **D,** Heatmap of nCounter Analysis System results for NIH:OVCAR-3 cells treated with carboplatin, cisplatin, and doxorubicin. Medium-only wells served as the nontreated control. The heatmap shows differential expression in genes involved in EMT (**C**) and ECM remodeling and ECM–receptor interaction (**D**) of each treatment group (*n* = 3).

We found a significant increase in the gene expression of *CLDN6*, *CD44*, and *TGFβ1* in NIH:OVCAR-3 cells treated with carboplatin and cisplatin (Fig. 5A). However, these gene expression changes were not observed in cells treated with doxorubicin and paclitaxel (Fig. 5A). The induction of *CLDN6* and *CD44* gene expression by carboplatin treatment was also observed in ovarian cancer cells (COV318 and COV362) and lung cancer cells (NCI-H1435; Fig. 5B).

Next, using the same samples described above, we examined the expression of genes related to EMT and cell matrix remodeling in NIH:OVCAR-3 cells treated with or without chemotherapeutic drugs. A heat map plot revealed that carboplatin upregulated EMT- and cell matrix remodeling–related genes, whereas cisplatin and doxorubicin had less or no impact on gene expression changes (Fig. 5C and D).

### TGFβ1 induces CLDN6^high^/CD44^high^ population in ovarian and lung adenocarcinoma cells

**Figure 6.**
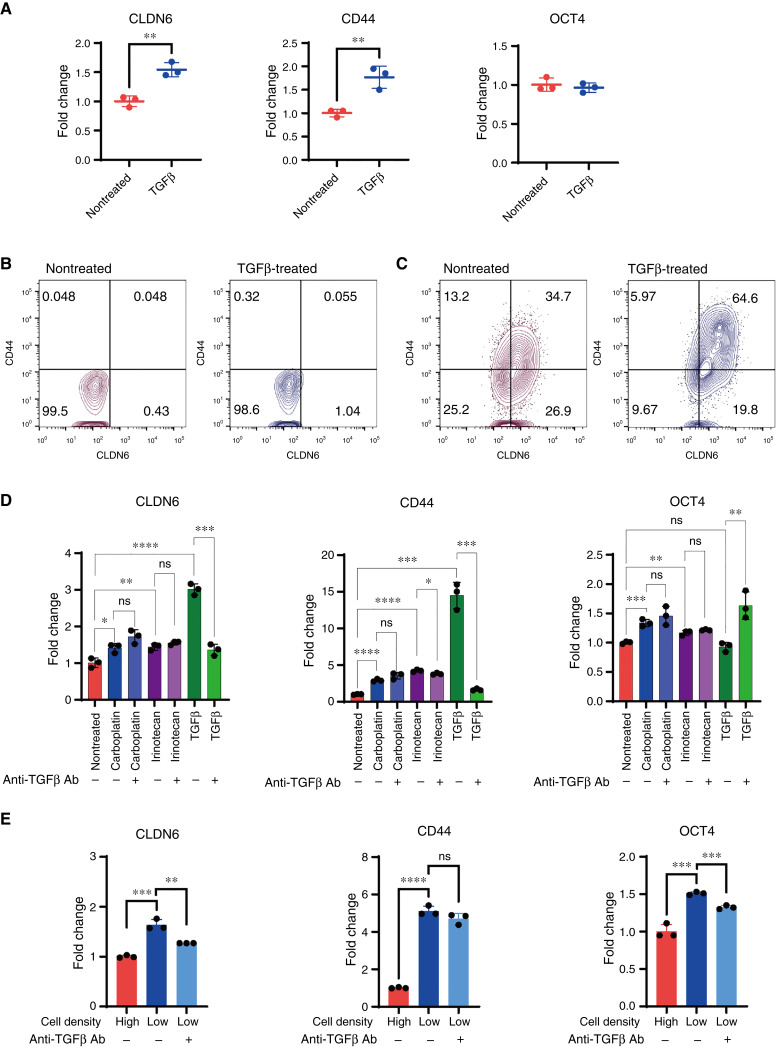
TGFβ1 increases CLDN6^high^/CD44^high^ population and is partially involved in low density–induced CLDN6 expression but not in cytotoxic agent–induced CLDN6 expression. **A,** qPCR analysis of *CLDN6*, *CD44*, and *OCT4* gene expression in NIH:OVCAR-3 cells treated with TGFβ1 (10 ng/mL) for 4 days. **B** and **C,** Flow cytometry plots of NIH:OVCAR-3 cells treated with or without TGFβ1 (10 ng/mL) for 4 days, assessed for the expression of CLDN6 and CD44 (**C**). Cells were also stained with isotype control as gating control (**B**). **D,** qPCR analysis of *CLDN6*, *CD44*, and *OCT4* gene expression in NIH:OVCAR-3 cells treated with carboplatin, irinotecan, or TGFβ1 in the presence or absence of anti-TGFβ antibody (100 μg/mL) for 72 hours. Medium-only wells served as the nontreated control. **E,** qPCR analysis of *CLDN6*, *CD44,* and *OCT4* gene expression in NIH:OVCAR-3 cells cultured at low density or high density in the presence or absence of anti-TGFβ antibody (100 μg/mL) for 6 days. Quantitative data are presented as the mean ± SD (*n* = 3). *P* values were determined by two-tailed Student *t* test. *, *P* < 0.05; **, *P* < 0.01; ***, *P* < 0.001; ****, *P* < 0.0001. Ab, antibody.

Uspregulation of *CLDN6* expression in cells grown at low density or treated with carboplatin was accompanied by increased *TGFβ1* expression (Figs. 3B and 5A), suggesting that TGFβ1 may directly contribute to CLDN6 expression. To investigate this hypothesis, we treated NIH:OVCAR-3 cells with TGFβ1 and conducted gene expression analysis. As expected, TGFβ1 treatment induced *CLDN6* and *CD44* mRNA expression, whereas *OCT4* expression remained unaffected (Fig. 6A). Flow cytometry analysis revealed an increase in the CLDN6^high^/CD44^high^ population following TGFβ1 treatment in NIH:OVCAR-3 (Fig. 6B and C), as well as other CLDN6-positive cell lines, COV362, COV413A, COV413B, and NCI-H1435 (Supplementary Fig. S9A–S9D and S9F–S9I), with the exception of OV-90 cells (Supplementary Fig. S9E and S9J).

Because we found that TGFβ1 directly increases CLDN6 expression, we further analyzed whether chemotherapy-induced TGFβ1promotes CLDN6 expression in an autocrine manner. As we have already confirmed, carboplatin significantly increased *CLDN6*, *CD44*, and *OCT4* mRNA expression (Fig. 6D). We also confirmed that irinotecan, a different type of chemotherapy drug from those previously evaluated, increased the expression of these genes (Fig. 6D). Notably, although the TGFβ-neutralizing antibody markedly inhibited TGFβ1-induced *CLDN6* and *CD44* expression, it did not attenuate either carboplatin- or irinotecan-induced upregulation of these genes (Fig. 6D). In low-density cultures, the TGFβ-neutralizing antibody partially inhibited low density–induced *CLDN6* and *OCT4* upregulation but had no effect on low density–induced *CD44* upregulation (Fig. 6E).

These results suggest that whereas EMT may be closely associated with the induction of the CLDN6^high^/CD44^high^ population in response to low cell density or chemotherapeutic treatment, this phenomenon does not seem to be solely mediated by an autocrine TGFβ response.

### Antitumor efficacy of SAIL66 in combination with chemotherapeutic drugs

The plasticity of *CLDN6* expression in response to carboplatin observed *in vitro* prompted us to investigate how carboplatin contributes to the antitumor efficacy of SAIL66 *in vivo*.

First, we compared the efficacy of SAIL66 when mice were treated with monotherapy or in combination with carboplatin, as indicated in the scheme shown in Supplementary Fig. S2A.

**Figure 7.**
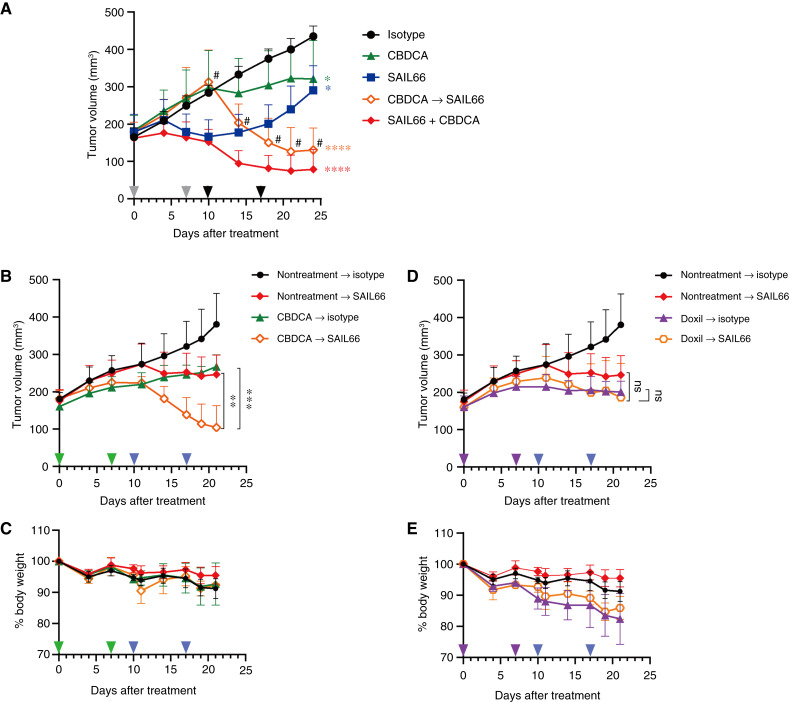
*In vivo* combination of SAIL66 and carboplatin shows synergistic antitumor efficacy. **A,** Combination therapy I: Antitumor efficacy of SAIL66 in combination with carboplatin in NIH:OVCAR-3 tumor–bearing huNOG mice. Mean tumor growth curves are shown according to the type of treatment indicated in Supplementary Fig. S2A (*n* = 5 per group). In the sequential group (orange line), one mouse showed body weight reduction on day 7 and was euthanized on day 9. Therefore, the data for this group consisted of 4 mice from day 10 (*n* = 4 indicated as #). Statistical differences in tumor volume at the endpoint were assessed between control and drug-administered groups using Dunnett *t* test with a significance level of 5% (two-sided). *, *P* < 0.05; ****, *P* < 0.0001. **B–E,** Combination therapy II: Efficacy and safety of SAIL66 in combination with cytotoxic agents, carboplatin (**B** and **C**) and doxil (**D** and **E**), in a sequential treatment regimen as indicated in Supplementary Fig. S2B (*n* = 5). **B** and **C,** Mean tumor volume (**B**) and percentage of body weight (**C**) in mice treated with isotype control on days 10 and 17 (nontreatment → isotype), SAIL66 on days 10 and 17 (no-treatment → SAIL66), carboplatin on days 0 and 7 followed by isotype control (CBDCA → isotype), or SAIL66 (CBDCA → SAIL66) on days 10 and 17. **D** and **E,** Mean tumor volume (**D**) and percentage of body weight (**E**) in mice treated with isotype control on days 10 and 17 (nontreatment → isotype), SAIL66 on days 10 and 17 (nontreatment → SAIL66), doxil on days 0 and 7 followed by isotype control (doxil → isotype), or SAIL66 (doxil → SAIL66) on days 10 and 17. Statistical analysis was performed between monotherapy and combination treatment groups using two-tailed Student *t* test. **, *P* < 0.01; ***, *P* < 0.001; ns, not significant.

In huNOG mice bearing NIH:OVCAR-3 tumors, monotherapy with either carboplatin or low-dose SAIL66 (0.3 mg/kg) demonstrated limited to moderate efficacy (Fig. 7A). However, simultaneous administration of carboplatin and SAIL66 exhibited enhanced tumor growth inhibition (Fig. 7A). Notably, sequential treatment with SAIL66 following carboplatin pretreatment resulted in more rapid tumor shrinkage (Fig. 7A).

We further compared the combination effects of two chemotherapeutic agents, carboplatin and doxorubicin, in sequential treatment with SAIL66 in NIH:OVCAR-3 tumor-bearing huNOG mice, as indicated in the treatment scheme shown in Supplementary Fig. S2B. A synergistic therapeutic effect was observed when SAIL66 was administered after carboplatin treatment (Fig. 7B) without body weight loss (Fig. 7C). In contrast, doxorubicin, which failed to induce *CLDN6* expression *in vitro* (Fig. 5A), did not show a synergistic effect when combined with SAIL66 and resulted in significant body weight loss (Fig. 7D and E).

### Carboplatin treatment is associated with TME modifications

**Figure 8.**
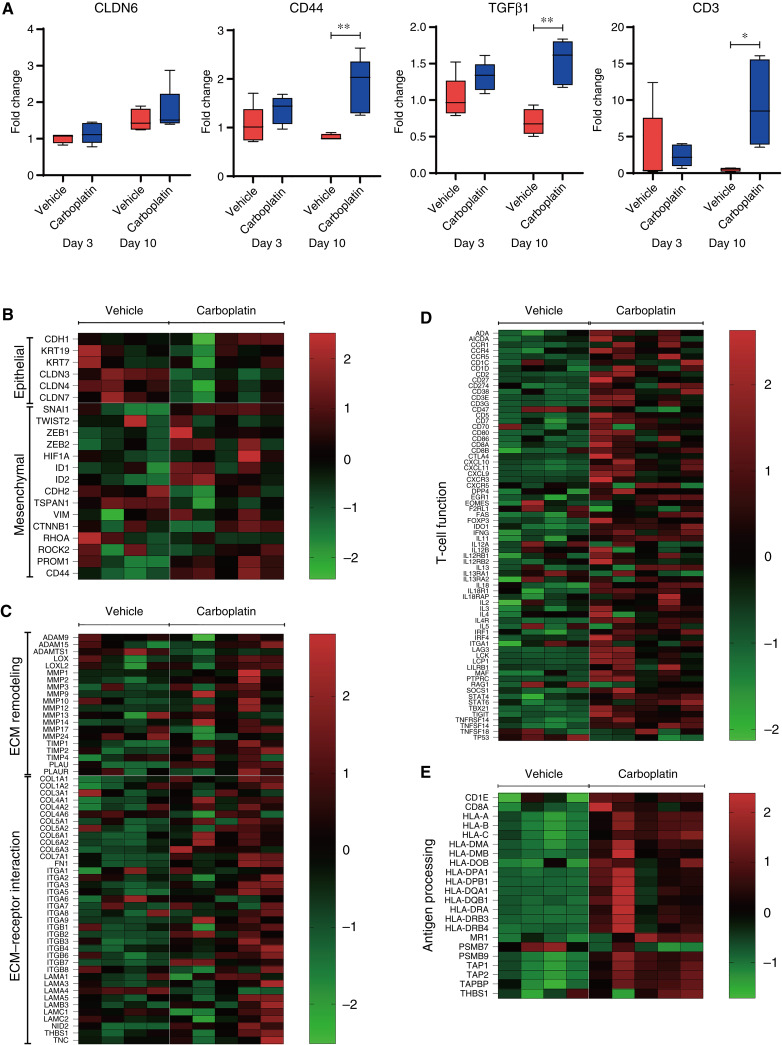
Carboplatin modulates the TME via EMT and enhances tumor immunity. **A,** qPCR analysis of *CLDN6*, *CD44*, *TGFβ1*, and *CD3* mRNA expression in huNOG mice bearing NIH:OVCAR-3 tumors quantified at day 3 and 10 after treatment with carboplatin at a dose of 30 mg/kg (blue) or vehicle (red). Quantitative data are presented as the mean ± SD (*n* = 5, except for vehicle group at day 10 where *n* = 4). *P* values were determined by two-tailed Student *t* test. *, *P* < 0.05; **, *P* < 0.01. **B–E,** Heatmap of nCounter Analysis System results for NIH:OVCAR-3 xenograft tumor in huNOG mice on day 10 after vehicle or carboplatin treatment. The heatmaps show differential expression of genes involved in EMT (**B**), ECM remodeling and ECM–receptor interaction (**C**), T-cell function (**D**), and antigen processing (**E**) between individual tumors treated with vehicle (*n* = 4) and carboplatin (*n* = 5).

To determine whether the carboplatin-induced EMT and CLDN6 expression observed *in vitro* also occur *in vivo*, we analyzed the impact of carboplatin treatment on NIH:OVCAR-3 tumor–bearing huNOG mice. qPCR analysis revealed a modest increase in *CLDN6* expression and significant increases in *CD44* and *TGFβ1* expression in tumors at 10 days after carboplatin administration compared with vehicle-treated controls (Fig. 8A). Furthermore, nCounter analysis of tumors at 10 days after administration confirmed the upregulation of EMT-related genes, as well as genes involved in ECM remodeling and ECM–receptor interactions following carboplatin treatment (Fig. 8B and C).

We next examined the impact of carboplatin on the tumor-immune microenvironment. qPCR analysis revealed a marked increase in *CD3* gene expression in tumors following carboplatin administration (Fig. 8A). nCounter analysis further confirmed the upregulation of multiple immune-related genes, including those associated with T-cell function and antigen processing, in carboplatin-treated tumors (Fig. 8D and E). Consistent with these gene expression profiles, histopathologic and IHC analysis demonstrated increased infiltration of CD3-positive cells in tumors 10 days after carboplatin administration (Supplementary Fig. S10A–S10D).

Overall, CLDN6 protein expression across the entire tumor showed no significant increase following carboplatin administration, as evidenced by H-scores from vehicle- and carboplatin-treated groups (Supplementary Table S3). However, a notable finding was the emergence of distinct CLDN6-positive cell clusters within tumors from the carboplatin-treated group, which were less frequently observed in vehicle-treated controls (Supplementary Fig. S10E–S10H; Supplementary Table S3).

## Discussion

In this study, we first noted the intratumoral heterogeneity of CLDN6-positive areas in EOC tissues. Next, we demonstrated the phenotypic plasticity of CLDN6 expression in ovarian cancer through *in vitro* culture experiment, revealing its dynamic regulation by cell density, chemotherapy, and TGFβ signaling. Specifically, we observed that CLDN6 expression is closely associated with EMT-related gene signatures. We then used spatial transcriptomic analysis of clinical specimens, which revealed phenotypically distinct CLDN6-positive regions with different gene signatures, revealing intratumoral heterogeneity. Finally, we translated these findings into a rational combination strategy to maximize the efficacy of CLDN6-targeted therapy.

Previous studies have demonstrated that knockdown of CLDN6 expression in gastric cancer cells decreased expression of SNAIL1, N-cadherin, and vimentin while increasing E-cadherin levels (28). Furthermore, it has been revealed that CLDN6 is functionally associated with tumor cell migration and invasion, as evidenced by gene silencing and forced expression experiments (28, 37–39, 52, 53). These studies suggested that elevated CLDN6 expressions in cancer cells may promote acquisition of malignant phenotypes during tumor progression through the process of EMT. In the present study, we demonstrated that spontaneous and reversible transitions between CLDN6-high and CLDN6-low status in the variety of CLDN6-positive ovarian cancer cell lines in response to different culture conditions coincided with alterations in EMT and cell matrix remodeling genes, which are closely associated with cancer cell migration and invasion (13, 54–56). Moreover, immunocytochemistry revealed variation in CLDN6 subcellular localization in cultured cancer cells depending on cell adhesion state, with cell–cell boundaries versus cytoplasm. These distinct localization patterns may reflect EMT-induced loss of polarity and disruption of cell–cell junctions, which represent critical steps in the invasion–metastasis cascade (57). Therefore, the spontaneous plasticity observed in CLDN6-positive cancer cells may enhance their metastatic potential, facilitating both dissemination from primary tumors and establishment of distant metastatic lesions. Further investigation of invasive potential and metastatic behavior of cancer cells under varying CLDN6 expression states would provide valuable insights into the biological significance of CLDN6 expression in ovarian cancer.

The density-dependent CLDN6 regulation observed in cancer cells may reflect fundamental aspects of stem cell differentiation processes (47, 58, 59). Indeed, it has been reported that CLDN6 expression in embryoid bodies exhibits temporal and spatial regulation in a density-dependent manner (44). Together with our findings, regulatory mechanisms governing CLDN6 expression in tumors may recapitulate those in normal development.

Among several cell lines tested *in vitro*, only OV-90 cells were minimally affected by cell density variations or TGFβ treatment. However, CLDN6 expression in both NIH:OVCAR-3 and OV-90 cells was substantially reduced in transplanted tumors compared with *in vitro*–cultured cells. These findings suggest that OV-90 cells exhibit plasticity not in simple cell monolayer cultures but rather when exposed to the complex TME architecture. Such architectural constraints impose alterations in cell polarity and morphology, resulting in modified mechanical and biochemical signals and subsequent changes in cell-to-cell communication. Collectively, our data indicate that CLDN6-positive cancer cells represent a highly plastic cell population with remarkable adaptive capabilities, which may explain the heterogenous CLDN6 expression observed in clinical tumors.

Our GeoMx spatial transcriptomics analysis revealed distinct gene expression patterns in CLDN6-positive cells between solid areas and dispersed small cluster areas within clinical specimens. Small clusters showed elevated EMT- and ECM-related genes compared with solid areas, likely corresponding to low-density cultures *in vitro* and representing a more invasive and aggressive cell population. Whereas higher CLDN6 expression correlates with worse prognosis across multiple cancer types (22, 24–26, 28–30, 32), focusing on phenotypic differences within CLDN6-positive clusters may provide more robust prognostic biomarkers than conventional IHC scoring alone. However, we acknowledge that the small sample size and lack of patient survival data (due to the use of commercial samples) limits generalizability. These findings should be considered preliminary and hypothesis-generating, requiring validation in larger, independent cohorts.

We demonstrated that TGFβ1 treatment increased the CLDN6/CD44-high cell population. As it is well established that TGFβ1 induces EMT and stem-like properties in cancer cells (16), and TGFβ signaling is linked to ovarian cancer progression (60, 61), TGFβ1 might play a key role in regulating CLDN6 expression during tumor development. Intriguingly, TGFβ blocking antibody failed to prevent CLDN6 and CD44 upregulation following chemotherapy treatment. This finding suggests the existence of alternative regulatory mechanisms beyond TGFβ signaling when tumors are treated with chemotherapy. One plausible pathway involves DNA damage response (DDR) and ATM activation (62). In our study, platinum-based agents and irinotecan strongly induced EMT. Recent studies have shown that these agents trigger DDR and ATM activation (62), which in turn stabilizes EMT transcription factors such as ZEB1 and SNAIL, leading to dynamic regulation of EMT (63, 64). However, our study did not comprehensively analyze these mechanisms in detail, and further investigation of other potential signaling pathways, including other potential signaling pathways, represents an important area for future research.

Our *in vivo* studies demonstrated that carboplatin pretreatment was associated with enhanced SAIL66 efficacy, accompanied by increased CLDN6 expression and alterations in immune cell infiltration within the TME. Carboplatin-induced modifications in the tumor-immune microenvironment are supported by previous reports analyzing HGSC samples collected before and after neoadjuvant chemotherapy (NACT). One study demonstrated a significant increase in CD8^+^ T-cell density after NACT (65), whereas another report demonstrated that NACT induced T-cell infiltration accompanied by spatially confined T-cell exhaustion (66). The antitumor efficacy of TCEs is dependent on baseline T-cell density within the TME (67). Building upon these established observations, our study provides novel insights by specifically examining how carboplatin-induced TME changes relate to CLDN6-targeted immunotherapy. We observed that carboplatin treatment was associated with upregulation of CLDN6-positive clusters in the TME as well as modulation of EMT-related markers and enhanced T-cell infiltration. These coordinated changes correlated with improved SAIL66 efficacy in our preclinical models, suggesting that chemotherapy-induced TME modifications may create a more permissive environment for CLDN6-targeted TCEs.

We acknowledge several limitations that warrant further investigation.

First, regarding mechanistic relationships between EMT and density- or chemotherapy-induced CLDN6 upregulation, although we established their correlations, direct causal relationships have not been experimentally validated. Future functional studies using knockdown or forced expression of EMT transcription factors would be necessary to determine whether they are necessary for CLDN6 upregulation.

Second, concerning the synergy mechanism of carboplatin on SAIL66 efficacy, it is important to emphasize that our current data establish correlations between TME changes by carboplatin and therapeutic synergy of SAIL66 rather than definitive causal relationships. Direct causal relationships require further experimental validation. Future studies using conditional knockout models or selective inhibitors of specific TME components would help establish causality and identify the critical mediators of this synergistic effect.

Third, regarding clinical validation, our *in vivo* study primarily utilized established cell lines, which do not fully recapitulate the molecular heterogeneity and complex TME of clinical ovarian cancers. Patient-derived xenograft models would provide more clinically relevant platforms for validating SAIL66 efficacy.

Fourth, our analysis of clinical specimens was limited. Although our observations of CLDN6 heterogeneity are consistent with prior reports (22, 25) and provide the first spatial transcriptomic characterization of molecular differences between CLDN6-positive regions, the small sample size limits statistical power and generalizability. We are currently analyzing additional clinical specimens to validate and extend these findings. Additionally, although the *in vitro* cell density model is informative for studying intrinsic cellular plasticity, it does not fully recapitulate the complex spatial organization, hypoxic gradients, stromal interactions, and other microenvironmental factors present in clinical tumors. Therefore, the *in vitro* findings should be interpreted as complementary mechanistic data that, together with our spatial transcriptomic analysis of clinical specimens, suggest potential mechanisms underlying CLDN6 heterogeneity.

Finally, the molecular mechanisms underlying TGFβ-independent, chemotherapy-induced CLDN6 upregulation remain incompletely characterized. Although DDR signaling represents one plausible pathway, comprehensive omics-based approaches following carboplatin treatment are necessary to identify all relevant mechanisms and represent an important direction for future research.

Our study comprehensively characterizes CLDN6 expression plasticity in ovarian cancer, in response to cell density, TGFβ stimuli, and chemotherapy, revealing association with EMT. Clinical specimens show substantial heterogeneity with distinct molecular signatures. Carboplatin pretreatment correlates with CLDN6 upregulation, TME modifications, and enhanced SAIL66 efficacy. These findings establish foundations for optimizing CLDN6-targeted strategies and have important clinical implications for designing rational combination approaches in CLDN6-positive ovarian cancer, particularly in the context of platinum-based chemotherapy regimens that remain the standard of care, by leveraging therapy-induced plasticity for precision cancer immunotherapy.

## Supplementary Material

Supplementary Table S1 Cell line list used in the study

Supplementary Table S2 H scores of CLDN6 in ovarian cancer tissues

Supplementary Table S3 CLDN6 expression in xenograft tumors following vehicle or carboplatin treatment

Supplementary Fig S1 Representative gating strategy for flow cytometry analysis of CLDN6 and CD44 expression

Supplementary Fig S2 Experimental scheme for in vivo studies

Supplementary Fig S3 Validation of anti-CLDN6 antibody specificity for immunohistochemistry

Supplementary Fig S4 Flow cytometry analysis of CLDN6 expression in cancer cell lines

Supplementary Fig S5 CLDN6 and Ki-67 expression in NIH:OVCAR-3 cells at different cell densities

Supplementary Fig S6 Isotype control staining for NIH:OVCAR-3 cells

Supplementary Fig S7 Quantification of CLDN6, CD44, and CLDN3 expression in NIH:OVCAR-3 cells

Supplementary Fig S8 Flow cytometry analysis of CLDN6 and CD44 expression in cancer cell lines cultured at different cell densities

Supplementary Fig S9 Flow cytometry analysis of CLDN6 and CD44 expression in cancer cell lines following TGFβ treatment

Supplementary Fig S10 Histopathological and immunohistochemical analysis of NIH:OVCAR-3 xenograft tumors following vehicle or carboplatin treatment

## Data Availability

The NanoString data reported in this study are available in the NCBI Gene Expression Omnibus under accession number GSE315251, GSE315732, GSE315733, and GSE315735. The spatial transcriptomic data are available under the accession number GSE317400. Additional data are available from the corresponding author upon reasonable request.
