# Supplementary material for: CLDN6 Expression Plasticity in Ovarian Cancer: Insights into Therapeutic Optimization for CLDN6-Targeted Immunotherapy
Source: Cancer Res Commun. 2026 Feb 25;6(2):383–401. doi: 10.1158/2767-9764.CRC-25-0399 (PMC13138224; doi:10.1158/2767-9764.CRC-25-0399)
Supplement: Supplementary Table S3 — CLDN6 expression in xenograft tumors following vehicle or carboplatin treatment [file crc-25-0399_supplementary_table_s3_suppst3.docx]

**Supplementary Table S3.**
