# Supplementary material for: CLDN6 Expression Plasticity in Ovarian Cancer: Insights into Therapeutic Optimization for CLDN6-Targeted Immunotherapy
Source: Cancer Res Commun. 2026 Feb 25;6(2):383–401. doi: 10.1158/2767-9764.CRC-25-0399 (PMC13138224; doi:10.1158/2767-9764.CRC-25-0399)
Supplement: Supplementary Fig S1 — Representative gating strategy for flow cytometry analysis of CLDN6 and CD44 expression [file crc-25-0399_supplementary_fig_s1_suppsf1.docx]

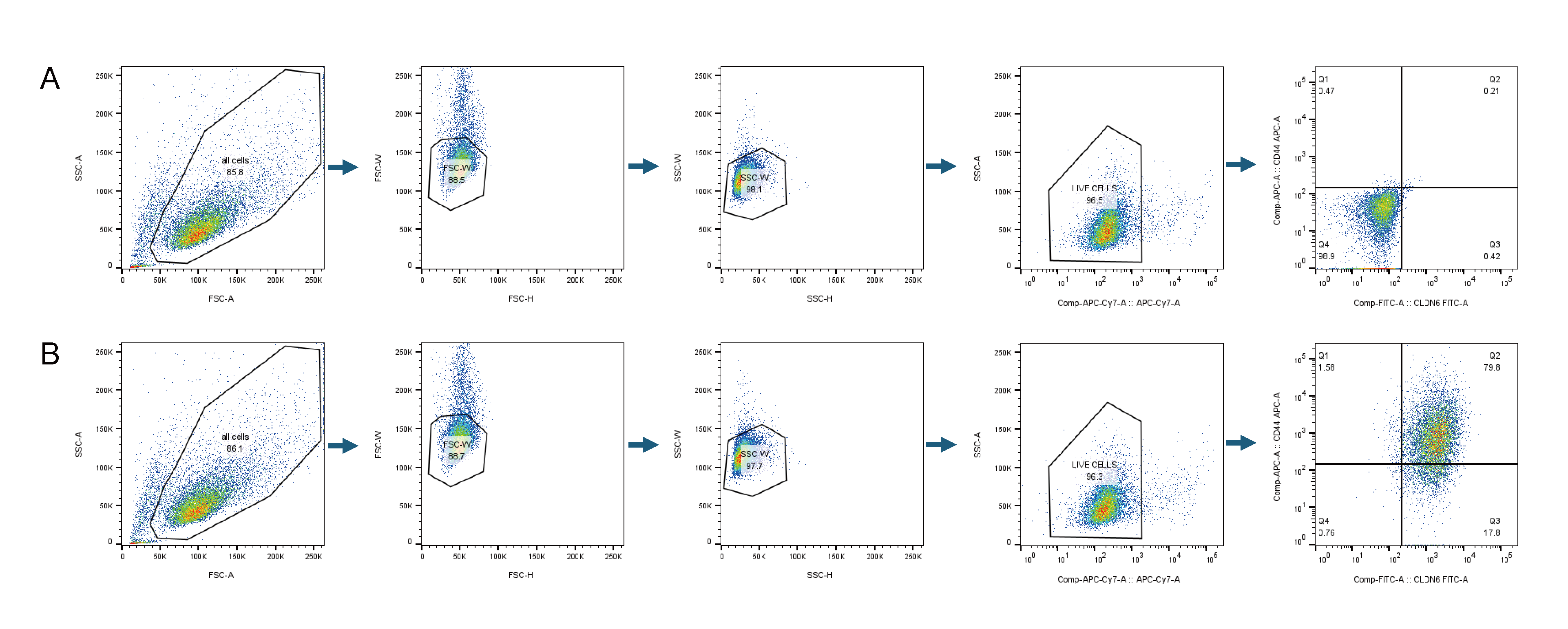


**Supplementary Fig S1. Representative gating strategy for flow cytometry analysis of CLDN6 and CD44 expression.** To identify CLDN6^high^/CD44^high^ population in cultured cells, the following gating sequence was used: FSC-A/SSC-A (size gating) → FSC-W/FSC-H (singlet gating) → SSC-W/SSC-H (singlet gating) → SSC-A/APC-Cy7 (live cell gating) → APC-CD44/FITC-CLDN6 **(B)**. Isotype-matched controls for CD44 and CLDN6 were used as gating controls **(A)**.
