# Supplementary material for: CLDN6 Expression Plasticity in Ovarian Cancer: Insights into Therapeutic Optimization for CLDN6-Targeted Immunotherapy
Source: Cancer Res Commun. 2026 Feb 25;6(2):383–401. doi: 10.1158/2767-9764.CRC-25-0399 (PMC13138224; doi:10.1158/2767-9764.CRC-25-0399)
Supplement: Supplementary Fig S2 — Experimental scheme for in vivo studies [file crc-25-0399_supplementary_fig_s2_suppsf2.docx]

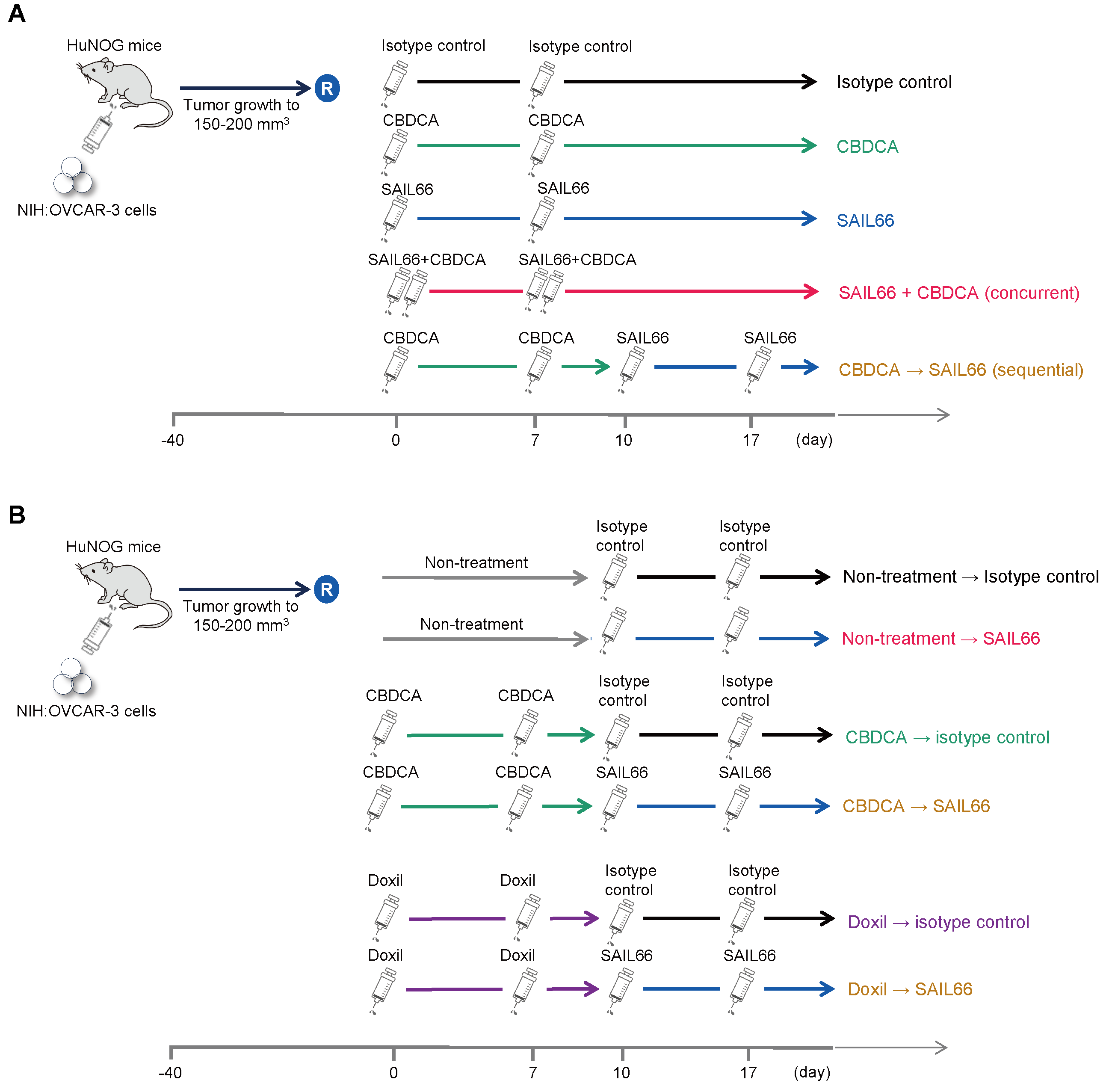


**Supplementary Fig S2. Experimental scheme for in vivo studies in the NIH:OVCAR-3 tumor-bearing huNOG model for tumor growth assays.** When tumors reached 150-200 mm^3^, mice were randomized into five **(Study I in A)** or six groups **(Study II in B)** and then received the treatments described below.

**(A)** Experimental scheme for combination therapy I. In the monotherapy groups, mice were treated with isotype control IgG (0.3 mg/kg), carboplatin (CBDCA, 30 mg/kg), or SAIL66 (0.3 mg/kg) alone on days 0 and 7. In the concurrent treatment group, mice received both SAIL66 and carboplatin on days 0 and 7. In the sequential treatment group, carboplatin was administered on days 0 and 7, followed by SAIL66 on day 10 and 17.

**(B)** Experimental scheme for combination therapy II. In the monotherapy group, mice were treated with isotype control IgG (0.3mg/kg) or SAIL66 (0.3mg/kg) on days 10 and 17. In the sequential treatment groups, mice were treated with two cycles of cytotoxic agents (30 mg/kg carboplatin or 10 mg/kg Doxil on days 0 and 7). Following pretreatment with cytotoxic agents, mice were treated with isotype control IgG (0.3 mg/kg) or SAIL66 (0.3 mg/kg) on days 10 and 17.
