# Supplementary material for: CLDN6 Expression Plasticity in Ovarian Cancer: Insights into Therapeutic Optimization for CLDN6-Targeted Immunotherapy
Source: Cancer Res Commun. 2026 Feb 25;6(2):383–401. doi: 10.1158/2767-9764.CRC-25-0399 (PMC13138224; doi:10.1158/2767-9764.CRC-25-0399)
Supplement: Supplementary Fig S3 — Validation of anti-CLDN6 antibody specificity for immunohistochemistry [file crc-25-0399_supplementary_fig_s3_suppsf3.docx]

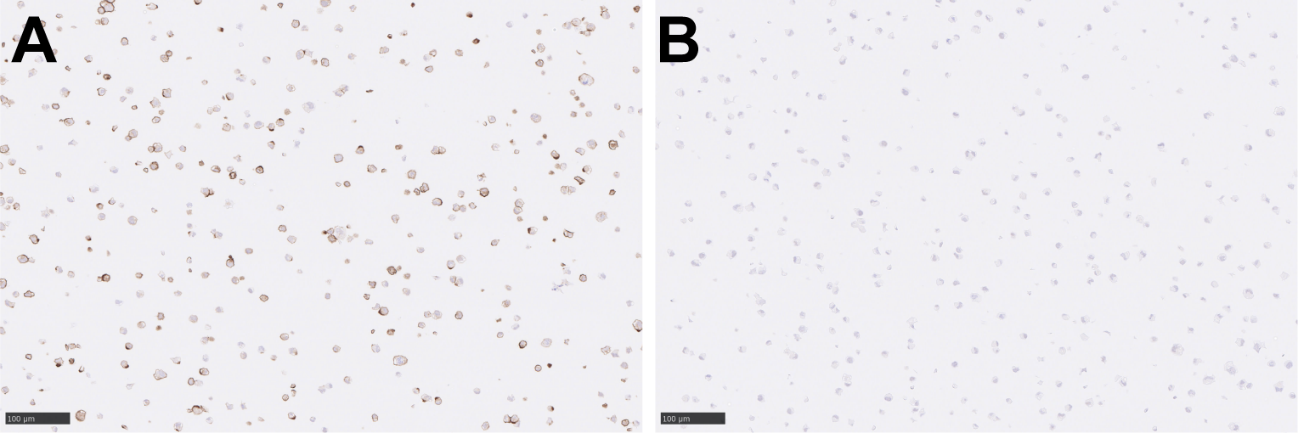


**Supplementary Fig S3. Validation of anti-CLDN6 antibody specificity for immunohistochemistry.** The specificity of an in-house generated anti-CLDN6 antibody for IHC (clone CDB0324ff) was confirmed using FFPE cell blocks by comparing SK-OV-3 cells transfected to express CLDN6 (SK-OV-3 Hi2C4) **(A)** with parental SK-OV-3 cells **(B)**.

Scale bar, 100 µm.
