# Supplementary material for: CLDN6 Expression Plasticity in Ovarian Cancer: Insights into Therapeutic Optimization for CLDN6-Targeted Immunotherapy
Source: Cancer Res Commun. 2026 Feb 25;6(2):383–401. doi: 10.1158/2767-9764.CRC-25-0399 (PMC13138224; doi:10.1158/2767-9764.CRC-25-0399)
Supplement: Supplementary Fig S4 — Flow cytometry analysis of CLDN6 expression in cancer cell lines [file crc-25-0399_supplementary_fig_s4_suppsf4.docx]

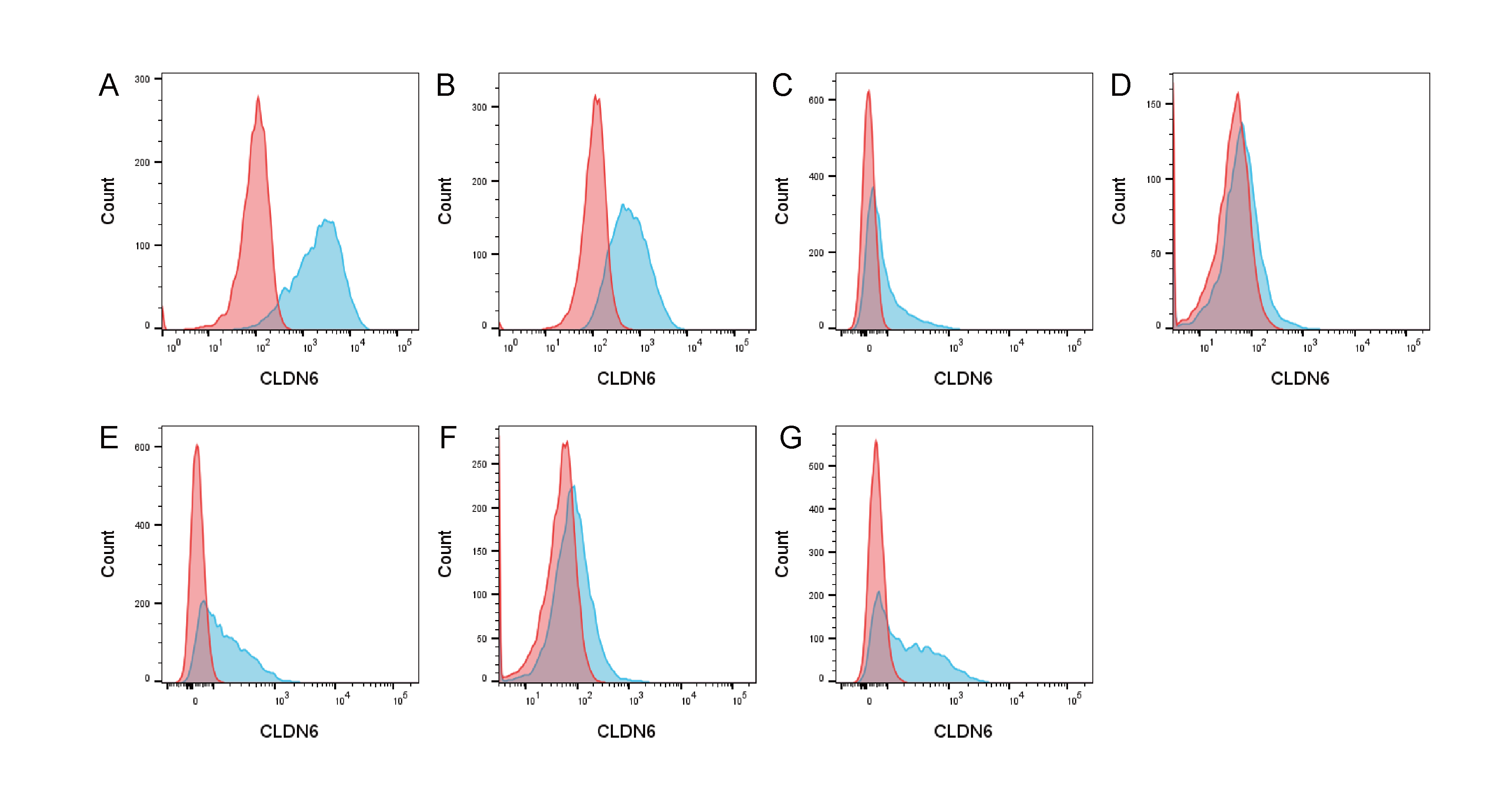


**Supplementary Fig S4.** **Flow cytometry analysis of CLDN6 expression in cancer cell lines.** OV-90 **(A)**, NIH:OVCAR-3 **(B)**, COV362 **(C)**, COV413A **(D)**, COV413B **(E)**, JHOS4 **(F)**, and NCI-H1435 **(G)** cells were harvested at subconfluent density and stained with anti-CLDN6 antibody. Red: isotype control, blue: CLDN6.
