# Supplementary material for: CLDN6 Expression Plasticity in Ovarian Cancer: Insights into Therapeutic Optimization for CLDN6-Targeted Immunotherapy
Source: Cancer Res Commun. 2026 Feb 25;6(2):383–401. doi: 10.1158/2767-9764.CRC-25-0399 (PMC13138224; doi:10.1158/2767-9764.CRC-25-0399)
Supplement: Supplementary Fig S5 — CLDN6 and Ki-67 expression in NIH:OVCAR-3 cells at different cell densities [file crc-25-0399_supplementary_fig_s5_suppsf5.docx]

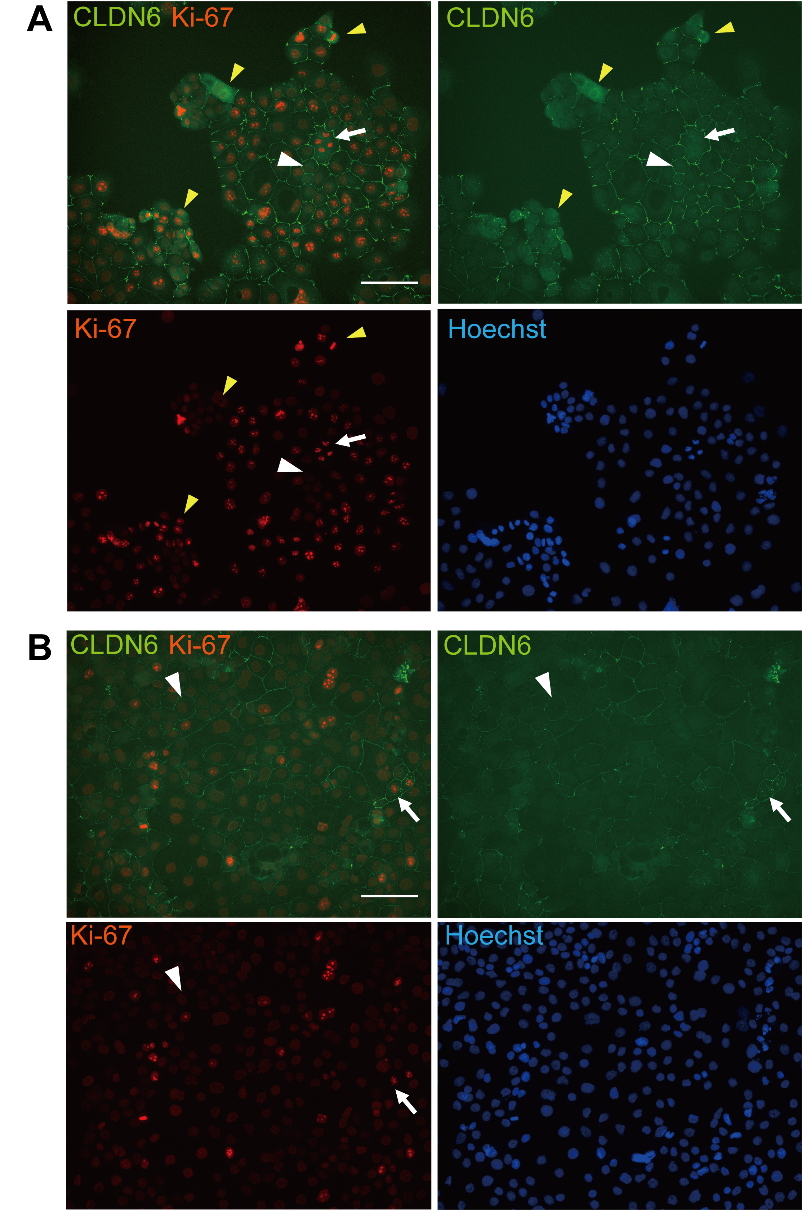


**Supplementary Fig S5. CLDN6 and Ki-67 expression in NIH:OVCAR-3 cells at different cell densities.** NIH:OVCAR-3 cells were cultured at low density **(A)** and high density **(B)** for 5 days. Expression of CLDN6 (green) and Ki-67 (red) in NIH:OVCAR-3 cells was evaluated by immunofluorescent double staining. CLDN6/Ki-67 double positive cells are indicated with white arrows, and CLDN6^+^/Ki-67^-^ cells are indicated with white arrowheads. Yellow arrowheads in cells at low cell density **(A)** indicate cells with intracellular CLDN6 expression. Nuclei were counterstained with Hoechst (blue). Sale bar, 100 µm.
