# Supplementary material for: CLDN6 Expression Plasticity in Ovarian Cancer: Insights into Therapeutic Optimization for CLDN6-Targeted Immunotherapy
Source: Cancer Res Commun. 2026 Feb 25;6(2):383–401. doi: 10.1158/2767-9764.CRC-25-0399 (PMC13138224; doi:10.1158/2767-9764.CRC-25-0399)
Supplement: Supplementary Fig S6 — Isotype control staining for NIH:OVCAR-3 cells [file crc-25-0399_supplementary_fig_s6_suppsf6.docx]

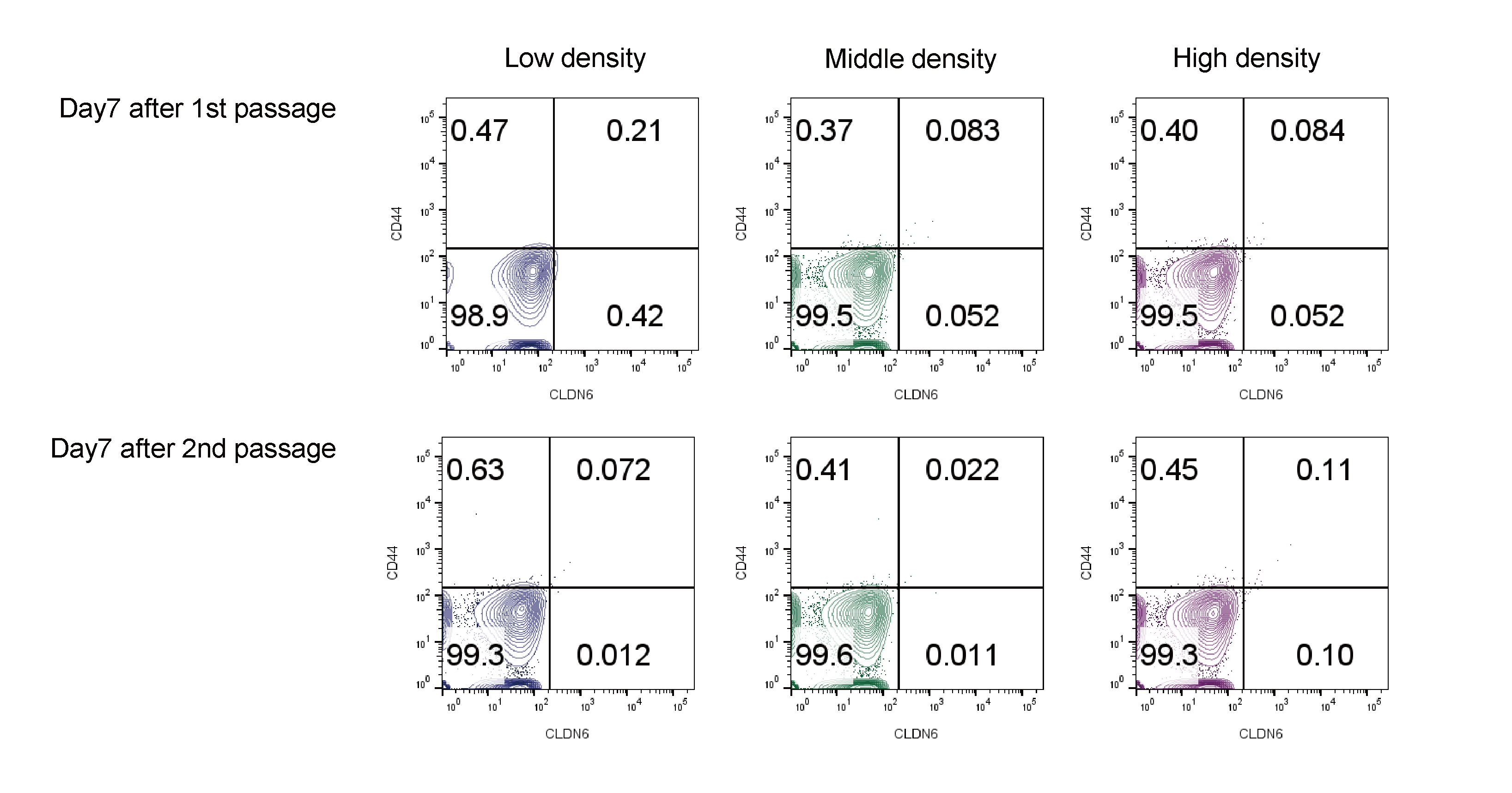


**Supplementary Fig S6. Isotype control staining for NIH:OVCAR-3 cells.** Isotype control staining for NIH:OVCAR-3 cells on day 7 after first and second passages at different cell densities, corresponding to **Fig. 3F.**
