# Supplementary material for: CLDN6 Expression Plasticity in Ovarian Cancer: Insights into Therapeutic Optimization for CLDN6-Targeted Immunotherapy
Source: Cancer Res Commun. 2026 Feb 25;6(2):383–401. doi: 10.1158/2767-9764.CRC-25-0399 (PMC13138224; doi:10.1158/2767-9764.CRC-25-0399)
Supplement: Supplementary Fig S7 — Quantification of CLDN6, CD44, and CLDN3 expression in NIH:OVCAR-3 cells [file crc-25-0399_supplementary_fig_s7_suppsf7.docx]

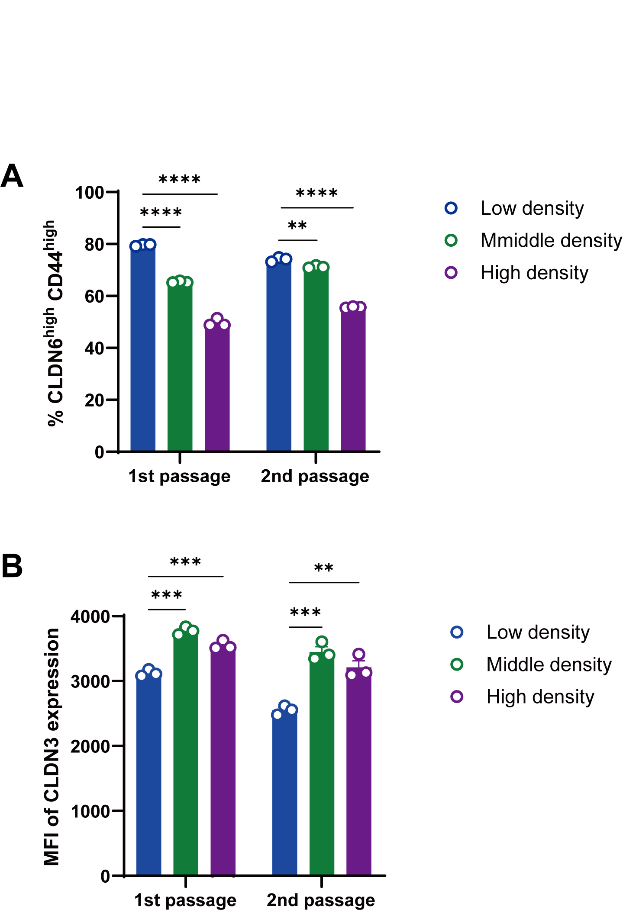


**Supplementary Fig S7. Quantification of CLDN6, CD44, and CLDN3 expression in NIH:OVCAR-3 cells.**

**(A)** Quantification of the CLDN6^high^ /CD44^high^ double-positive population (n=3 independent experiments).

**(B)** Quantification of median fluorescence intensity (MFI) of CLDN3 expression in NIH:OVCAR-3 cells after first and second passages at different cell densities, assessed by flow cytometry histogram (n=3 independent experiments).

Quantitative data are presented as mean ± SD. Two-tailed Student’s t-test. **, P <0.01; ***, P <0.001; ***, P <0.0001.
