# Supplementary material for: CLDN6 Expression Plasticity in Ovarian Cancer: Insights into Therapeutic Optimization for CLDN6-Targeted Immunotherapy
Source: Cancer Res Commun. 2026 Feb 25;6(2):383–401. doi: 10.1158/2767-9764.CRC-25-0399 (PMC13138224; doi:10.1158/2767-9764.CRC-25-0399)
Supplement: Supplementary Fig S8 — Flow cytometry analysis of CLDN6 and CD44 expression in cancer cell lines cultured at different cell densities [file crc-25-0399_supplementary_fig_s8_suppsf8.docx]

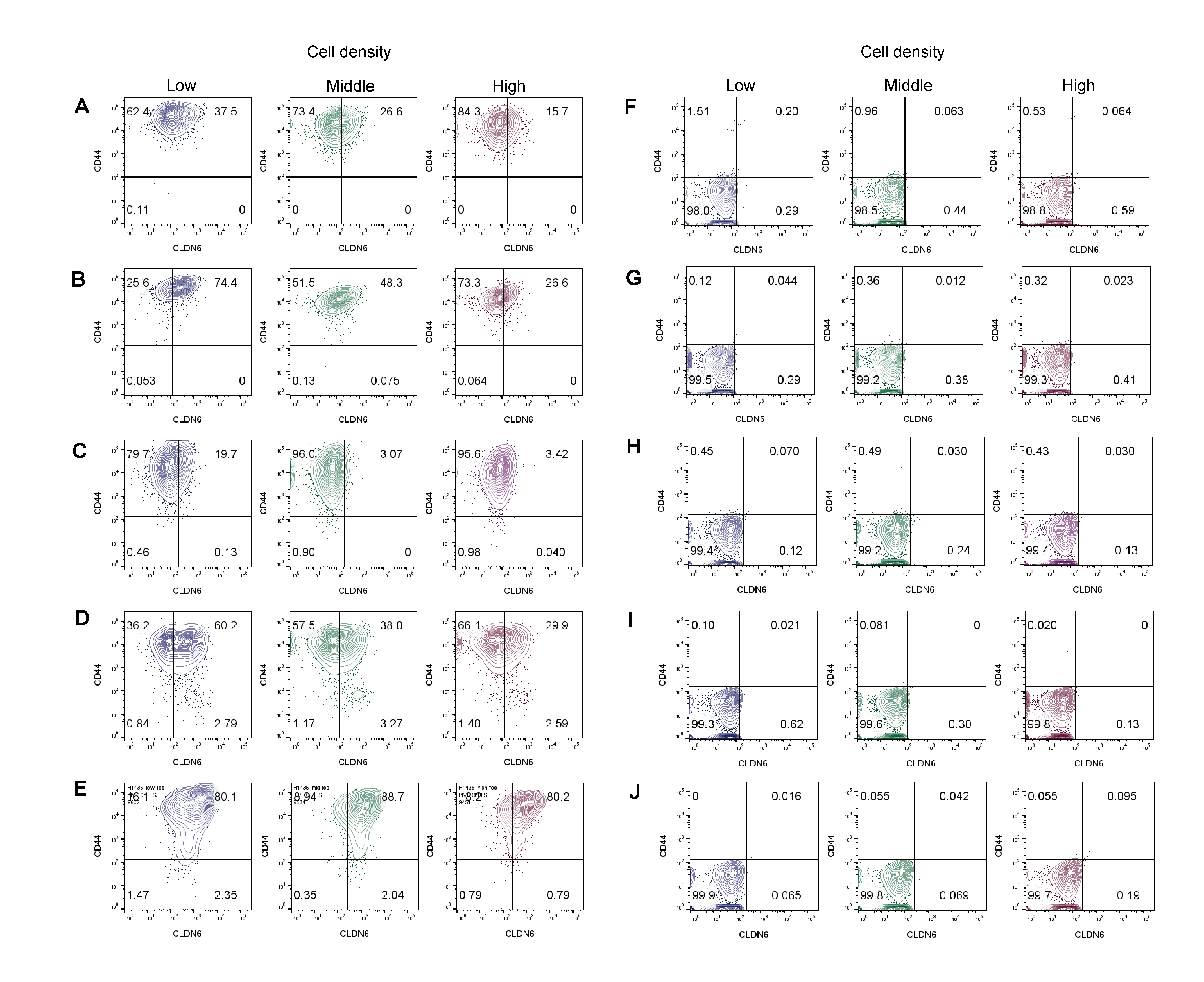


**Supplementary Fig S8. Flow cytometry analysis of CLDN6 and CD44 expression in cancer cell lines cultured at different cell densities. (A-E)** Flow cytometry plots of COV362 **(A)**, COV413B **(B)**, JHOS4 **(C)**, NCI-H1435 **(D)**, and OV-90 **(E)** cell lines cultured at different cell densities (from low to high) for 7 days, showing for CLDN6 and CD44 expression. **(F-J)** Isotype control staining for COV362 **(F)**, COV413B **(G)**, JHOS4 **(H)**, NCI-H1435 **(I)**, and OV-90 **(J)** cell lines corresponding to **(A-E)**.
