# Supplementary material for: CLDN6 Expression Plasticity in Ovarian Cancer: Insights into Therapeutic Optimization for CLDN6-Targeted Immunotherapy
Source: Cancer Res Commun. 2026 Feb 25;6(2):383–401. doi: 10.1158/2767-9764.CRC-25-0399 (PMC13138224; doi:10.1158/2767-9764.CRC-25-0399)
Supplement: Supplementary Fig S9 — Flow cytometry analysis of CLDN6 and CD44 expression in cancer cell lines following TGFβ treatment [file crc-25-0399_supplementary_fig_s9_suppsf9.docx]

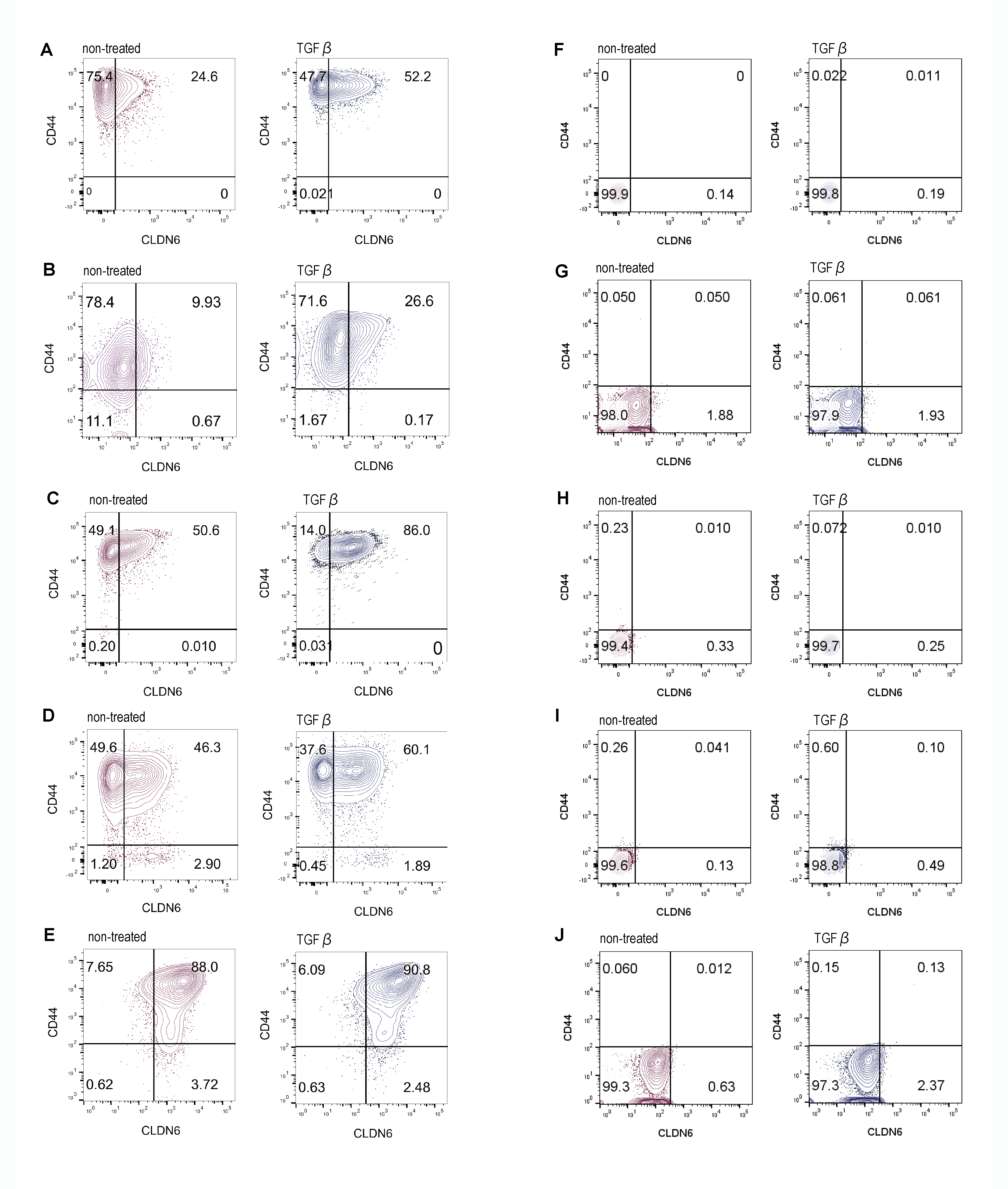


**Supplementary Fig S9. Flow cytometry analysis of CLDN6 and CD44 expression in cancer cell lines following TGFβ treatment. (A-E)** Flow cytometry plots of COV362 **(A)**, COV413A **(B)**, COV413B **(C)**, NCI-H1435 **(D)**, and OV-90 **(E)** cell lines treated with medium only (non-treated) and 10 ng/ml TGFβ for 4 days, showing CLDN6 and CD44 expression. **(F-J)** Isotype control staining for COV362 **(F)**, COV413A **(G)**, COV413B **(H)**, NCI-H1435 **(I)**, and OV-90 **(J)** cells with or without TGFβ treatment corresponding to **(A-E)**.
