# Supplementary material for: CLDN6 Expression Plasticity in Ovarian Cancer: Insights into Therapeutic Optimization for CLDN6-Targeted Immunotherapy
Source: Cancer Res Commun. 2026 Feb 25;6(2):383–401. doi: 10.1158/2767-9764.CRC-25-0399 (PMC13138224; doi:10.1158/2767-9764.CRC-25-0399)
Supplement: Supplementary Fig S10 — Histopathological and immunohistochemical analysis of NIH:OVCAR-3 xenograft tumors following vehicle or carboplatin treatment [file crc-25-0399_supplementary_fig_s10_suppsf10.docx]

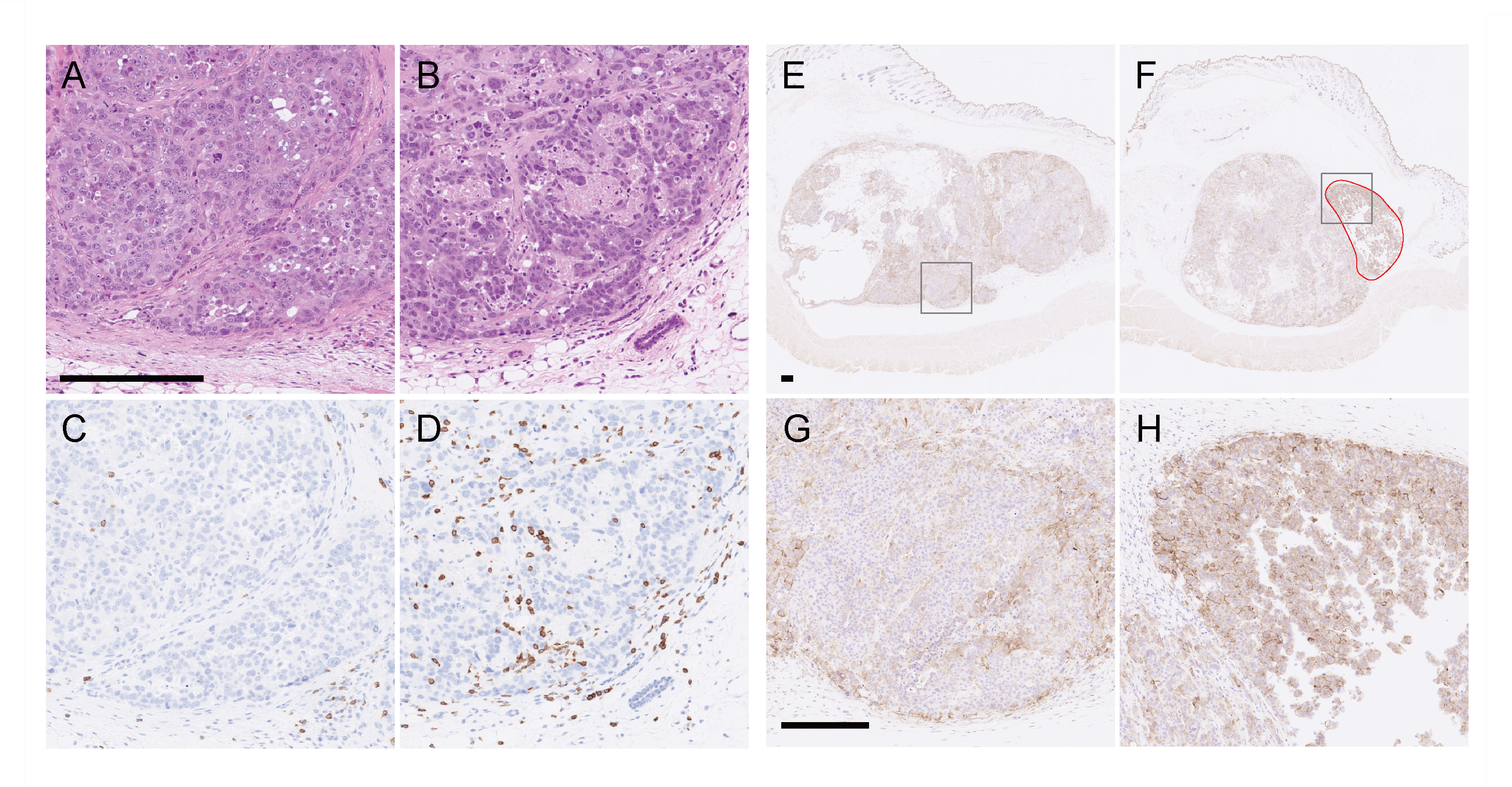


**Supplementary Fig S10. Histopathological and immunohistochemical analysis of NIH:OVCAR-3 xenograft tumors following vehicle or carboplatin treatment.** Pathological analysis of tumor tissue by H&E staining and IHC from NIH:OVCAR-3 xenograft tumors following vehicle (**A, C, E, and G**) or carboplatin administration at 30 mg/kg (**B, D, F, and H**).

**(A-D)** Representative images of H&E staining **(A and B)** and CD3 IHC staining **(C and D)**.

**(E-H)** Representative images of CLDN6 IHC staining in tumors from vehicle **(E and G)** and carboplatin-treated **(F and H)** mice. Red circle in **(F)** indicates a representative CLDN6-positive cell cluster with an area greater than 0.6 mm^2^. **(G and H)** Higher magnification images of the boxed regions shown in **(E)** and **(F)**, respectively.

Scale bars, 250 μm.
